# iPSC-derived cardiomyocytes from patients with myotonic dystrophy type 1 have abnormal ion channel functions and slower conduction velocities

**DOI:** 10.1038/s41598-021-82007-8

**Published:** 2021-01-28

**Authors:** Hugo Poulin, Aurélie Mercier, Mohammed Djemai, Valérie Pouliot, Isabelle Deschenes, Mohamed Boutjdir, Jack Puymirat, Mohamed Chahine

**Affiliations:** 1 CERVO Brain Research Centre, Quebec, QC Canada; 2 Department of Physiology and Cell Biology, Ohio State University, Columbus, OH USA; 3 Cardiovascular Research Program, VA New York Harbor Healthcare System, Brooklyn, New York, NY USA; 4 Department of Medicine, Cell Biology and Pharmacology, State University of New York Downstate Medical Center, New York, NY USA; 5 Depatrment of Medicine, NYU School of Medicine, New York, NY USA; 6 Unit of Human Genetics, Hôpital de l’Enfant-Jésus, CHU Research Center, Quebec, QC Canada; 7 Department of Medicine, Faculty of Medicine, Université Laval, Quebec, QC Canada

**Keywords:** Cell biology, Genetics, Molecular biology, Physiology, Stem cells, Cardiology, Diseases, Medical research, Pathogenesis

## Abstract

Cardiac complications such as electrical abnormalities including conduction delays and arrhythmias are the main cause of death in individuals with Myotonic Dystrophy type 1 (DM1). We developed a disease model using iPSC-derived cardiomyocytes (iPSC-CMs) from a healthy individual and two DM1 patients with different CTG repeats lengths and clinical history (DM1-1300 and DM1-300). We confirmed the presence of toxic RNA foci and mis-spliced *MBNL1/2* transcripts in DM1 iPSC-CMs. In DM1-1300, we identified a switch in the cardiac sodium channel *SCN5A* from the adult to the neonatal isoform. The down-regulation of adult *SCN5A* isoforms is consistent with a shift in the sodium current activation to depolarized potentials observed in DM1-1300. L-type calcium current density was higher in iPSC-CMs from DM1-1300, which is correlated with the overexpression of the Ca_V_1.2 transcript and proteins. Importantly, I_Na_ and I_CaL_ dysfunctions resulted in prolonged action potentials duration, slower velocities, and decreased overshoots. Optical mapping analysis revealed a slower conduction velocity in DM1-1300 iPSC-CM monolayers. In conclusion, our data revealed two distinct ions channels perturbations in DM1 iPSC-CM from the patient with cardiac dysfunction, one affecting Na^+^ channels and one affecting Ca^2+^ channels. Both have an impact on cardiac APs and ultimately on heart conduction.

## Introduction

Myotonic dystrophy type 1 or DM1, was first described in 1909 by Steinert^1^. It is a multi-systemic disease and is the most common adult form of muscular dystrophy^2^. DM1 is caused by the expansion of a CTG repeat in the 3′ untranslated region (3′-UTR) of a cAMP-dependent protein kinase (*DMPK,* for Dystrophia Myotonica Protein Kinase).

The disease affects multiple tissues and causes a variety of symptoms, including cardiac electrical abnormalities, myotonia, muscular dystrophy and neuropsychiatric disorder. The pathogenic mechanism involves an RNA gain-of-function in which DMPK repeat-containing transcripts accumulate in nuclei to form nuclear foci (ribonuclear inclusion). This process results in the sequestration and alteration in the functions of RNA-binding proteins involved in regulating RNA splicing^3^. The splicing regulation defect, or spliceopathy, has been linked to changes in the activities of two families of splicing regulators: CELF^4^ family and MBNL^5,6^ famil

Cardiac involvement, characterized by electrocardiographic (ECG) abnormalities, occurs in 80% of DM1 patients, often precedes the skeletal muscle involvement and causes a high incidence of sudden death (30%)^7^. The cardiac manifestations of DM1 include conduction defects in addition of ventricular and atrial arrhythmias^8^. Conduction and repolarization abnormalities have a prevalence of 40% and are the most commonly observed cardiac abnormalities in DM1 patients^9^. These manifest as a prolonged PR interval prolongation (atrioventricular block) and intraventricular conduction delays (left/right bundle branch block). A recent follow-up study of 855 DM1 patients reported 210 patient deaths (25%), including 32 sudden deaths (4%), 166 supraventricular arrhythmias developed over lifetime (19%), and 17 sustained ventricular tachyarrhythmias (2%), while permanent pacemakers were implanted in 181 patients (21%). There is a correlation between the rates of progression of conduction defects and the size of CTG repeats^10^. To prevent fatal cardiac electrical events, the implantation of an ICD is required in 3–22% of cases^11^. In 2015/16, two studies conducted in mice made a link between alterations in cardiac sodium channels and the electrical dysfunction in DM1^12,13^. However, no data are available regarding the potential role of Na^+^ and Ca^2+^ channels in the electrical abnormalities seen in DM1 patients using human cardiomyocytes relevant to a specific pathology such as DM1.

To gain a better understanding of the pathophysiology of DM1 cardiac electrical phenotype, we take advantage of the human induced pluripotent stem cells (hiPSC). DM1 patient-specific hiPSC were used in this study and differentiated into cardiomyocytes (hiPSC-CM) to model the disease. Electrical and biochemical properties of iPSC-CMs from a DM1 patient with severe cardiac manifestation were compared to iPSC-CMs from both, a DM1 patient without cardiac manifestation and from a healthy individual.

## Methods

Detailed descriptions are presented in the Online Supplementary Methods.

### Production, derivation, culture and characterization of patient-specific hiPSCs

Skin biopsies from a healthy donor and two DM1 patients, one with 300 CTG repeats (DM1-300) and one with 1300 CTG repeats (DM1-1300) were collected with informed consent and in accordance with the guidelines of CHU de Québec-Université Laval Research Ethics Committee (PEJ 560). The dermal fibroblasts were reprogrammed and characterized at the LOEX core facility (Quebec, QC, Canada) using the OCT3/4, SOX2, KLF4, and C-MYC factors and the non-integrating Sendai virus method. For each individual, we used one cell line of iPSC during this study, thus three cell lines of iPSC were used in total. The local ethics committee (Comité d’éthique du centre intégré universitaire de santé et de services sociaux (CIUSSS) de Ia capitale nationale de Québec), approved the study protocol (2019–1734: DM1).

### Patient-specific hiPSC cardiomyocyte differentiation

Three iPSC cell lines, one from each individual, were used to perform all the experiments. iPSCs were grown on hESC-qualified Matrigel in mTeSR1 media (StemCell Technologies) and were routinely passaged using dispase. The iPSCs were differentiated into cardiomyocytes using the Wnt signaling using a series of reagents in a time-controlled manner based on published protocol^14,15^. The experiments on iPSC-CM were done using iPSC-CM from independent differentiations. Thus, many cell differentiations were carried throughout the study to generate the iPSC-CMs.

### ASPCR analyses

RNA was extracted from iPSC-CMs on day 30 of differentiation from three independent differentiation experiments using Trizol (Life Technologies, CA, USA). Alternative splicing analyses by end-point RT-PCR (ASPCR) were carried out at the Université de Sherbrooke RNomics Platform with a screening of 177 alternative splicing events.

### Quantitative real-time PCR analysis

RNA was extracted from iPSC-CMs on day 30 or day 60 of differentiation using Trizol, and cDNA were synthesized using the Protoscript II First Strand cDNA synthesis kit protocol (NEB, Ipswich, MA, USA). qPCR assays were performed using SYBR green I detection dye on an LC480 platform (Roche, Basel, Switzerland) using the vendor’s specifications.

### Immunofluorescence staining

iPSC-CMs were dissociated from monolayers using TrypLE Express (ThermoFisher Scientific, Saint-Laurent, QC, Canada) and were plated on Matrigel-treated 13-mm TC coverslips (Sarstedt, Saint-Léonard, QC, Canada). The cells were fixed, permeabilized and were then incubated with primary and secondary antibodies. The cells were observed using a Zeiss LSM confocal microscope equipped with a 63 × oil objective and the appropriate laser and filters.

### RNA fluorescence in situ hybridization (FISH)

CUG-containing foci were detected in iPSC-CMs grown on coverslips using a 5′-Cy3-labeled (CAG)_5_ peptide nucleic acid probe (PNA Bio, Thousand Oaks, CA, USA) as described previously^16^.

### Western blotting

Protein extractions from hiPSC-CMs were carried out on day 30 of maturation, as described previously^17^. Western blots were performed on 20 μg of total protein extract in denatured condition.

### Electrophysiology

Patch clamp experiments on iPSC-CMs were performed at room temperature on dissociated hiPSC-CMs at days 30–40 post-differentiation, as described previously^18,19^. Currents and voltages were recorded in whole cell configuration using an Axopatch 200 amplifier and pClamp software (Molecular Devices, San José, CA, USA).

### Optical mapping of iPSC-CMs monolayers

Optical mapping experiment were conducted at 37 °C on reconstituted iPSC-CM monolayers at day 30 days post-differentiation. The cells were stained with di-4-ANEPPS and the membrane voltage was mapped with a high-speed CMOS N256 camera (MiCAM03, SciMedia, Costa Mesa, CA, USA) using a 530 nm green LED light source (LEX2-LZ4-G, SciMedia).

### Statistics

All statistical analysis was performed using the software Prism8 (GraphPad, San Diego, CA, USA). Normality of all data sets were determined using the Kolmogorov–Smirnov or D’Agostino-Pearson test. Multiple comparisons between CTRL, DM1-300 and DM1-1300 groups were performed using ANOVA and Tukey’s post hoc test.

## Results

### Clinical evaluation

#### Patient 1: DM1-1300

The index patient was a 27-year-old male with an infantile (onset 2–10 years) form of DM1 with approximately 1300 CTG triplet repeats, based on an evaluation using his circulating leukocytes. He had a pacemaker installed in 2013 because of conduction abnormalities including a third-degree atrioventricular (AV) block. ECG examinations revealed an increase in the PR interval over four years: 182 ms in 2009, 192 ms in 2011, and > 200 ms in 2013 (Fig. S1). He also had cognitive problems.

His father and brother also had a pacemaker implanted due to conduction abnormalities.

#### Patient 2: DM1-300

The index DM1 patient was a 38-year-old male with an adult form of the disease (onset at age 20–40 years). He was not related to patient 1. He had approximately 300 CTG triplet repeats and no cardiac abnormalities. He had normal PR and QRS intervals of 184 ms and 96 ms, respectively. He also developed cognitive problems.

#### Healthy individual: CTRL

This individual was a healthy thirty-year-old man with no signs of DM1. He had been genotyped with five CTG triplet repeats which is considered in the normal range. He has no relationship with the two DM1 patients.

### iPS-derived cardiomyocytes from DM1 exhibit typical disease phenotypes

We have generated three iPS cell lines, one for each individual described above.: CTRL, DM1-1300 and DM1-300. These iPS cell lines were then differentiated into cardiomyocytes (iPSC-CMs) through the canonical Wnt pathway to investigate the cardiac electrical abnormalities seen in DM1 (Fig. S2). In this work, the iPSC-CM from the patient 2 (DM1-300) was used as a control to discard phenotype not-related to cardiac dysfunction, as this patient has no manifestation of cardiac defects.

**Figure 1:**
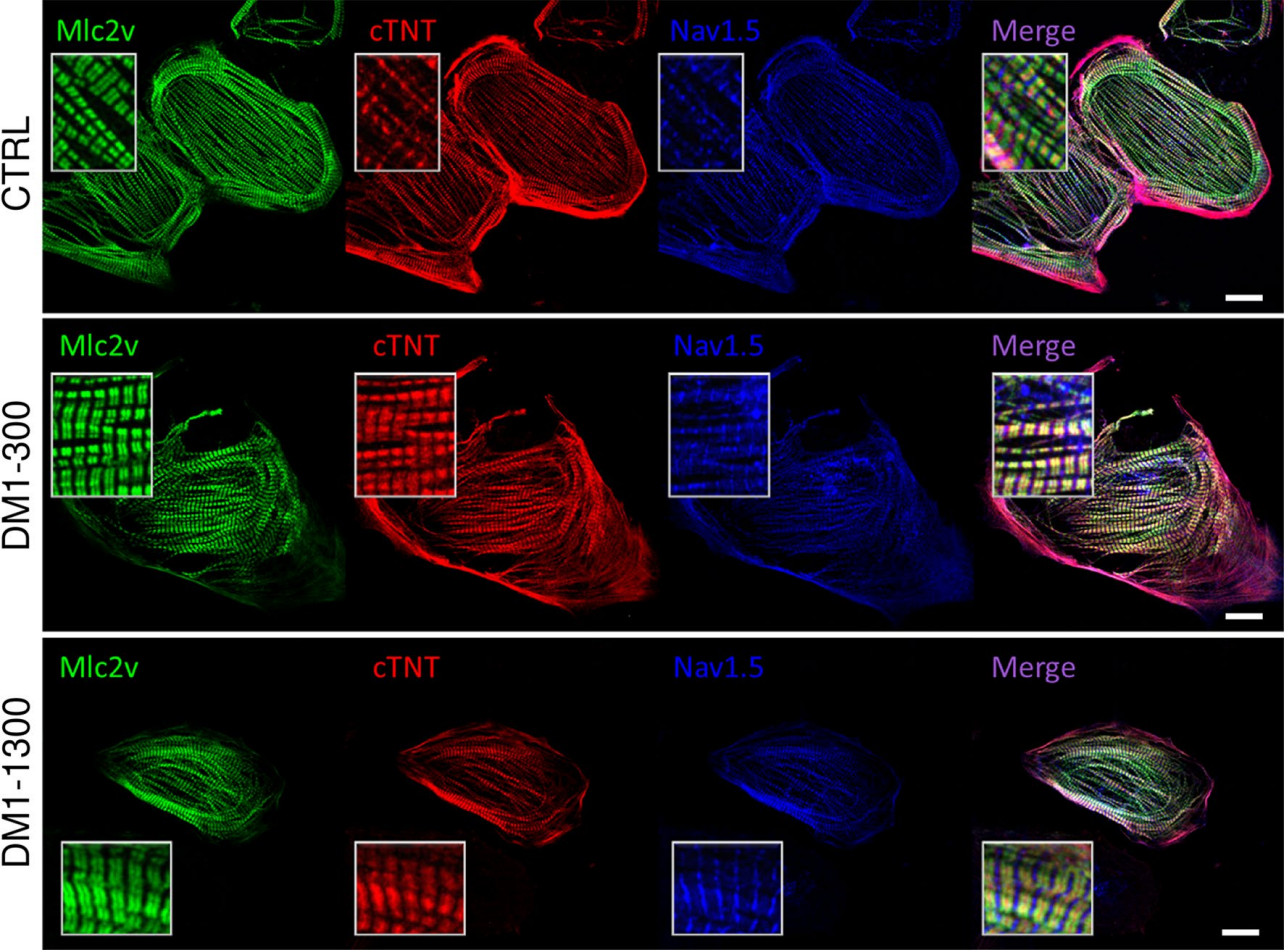
Immunofluorescence staining of cardiac markers in iPSC-CMs after 30 days of maturation. iPSC-CMs from the CTRL individual and the DM1-300 and DM1-1300 patients showed organized contractile proteins, including myosin light chain2v (mlc2v, green staining), cardiac troponin T (cTnT, red staining), and the cardiac sodium channel (NaV1.5, blue staining). The last column shows merged images from mlc2v, cTnT, and NaV1.5. The insets show a zoom-in of the stained proteins. Scale bar: 20 µM. Images were acquired using Zeiss ImagerM2 LSM confocal microscope and processed with ZEN software (Zeiss).

To characterize the iPS-CMs morphologies, the sarcomeric organization was revealed by immunofluorescence staining for myosin light chain 2v (MLC2v), cardiac troponin T (cTnT), and the cardiac sodium channel Na_V_1.5. The confocal microscopy images show normal organization of the contractile proteins as well as Na_V_1.5 channels distributed in all three iPSC-CMs (Fig. 1). Using mcl2v and α-actinin staining, the length of the sarcomere was measured, namely the distance between two α-actinin segments, and was similar (~ 1.9 µm) in CTRL, DM1-300 and DM1-1300 (Fig. S3).

**Figure 2:**
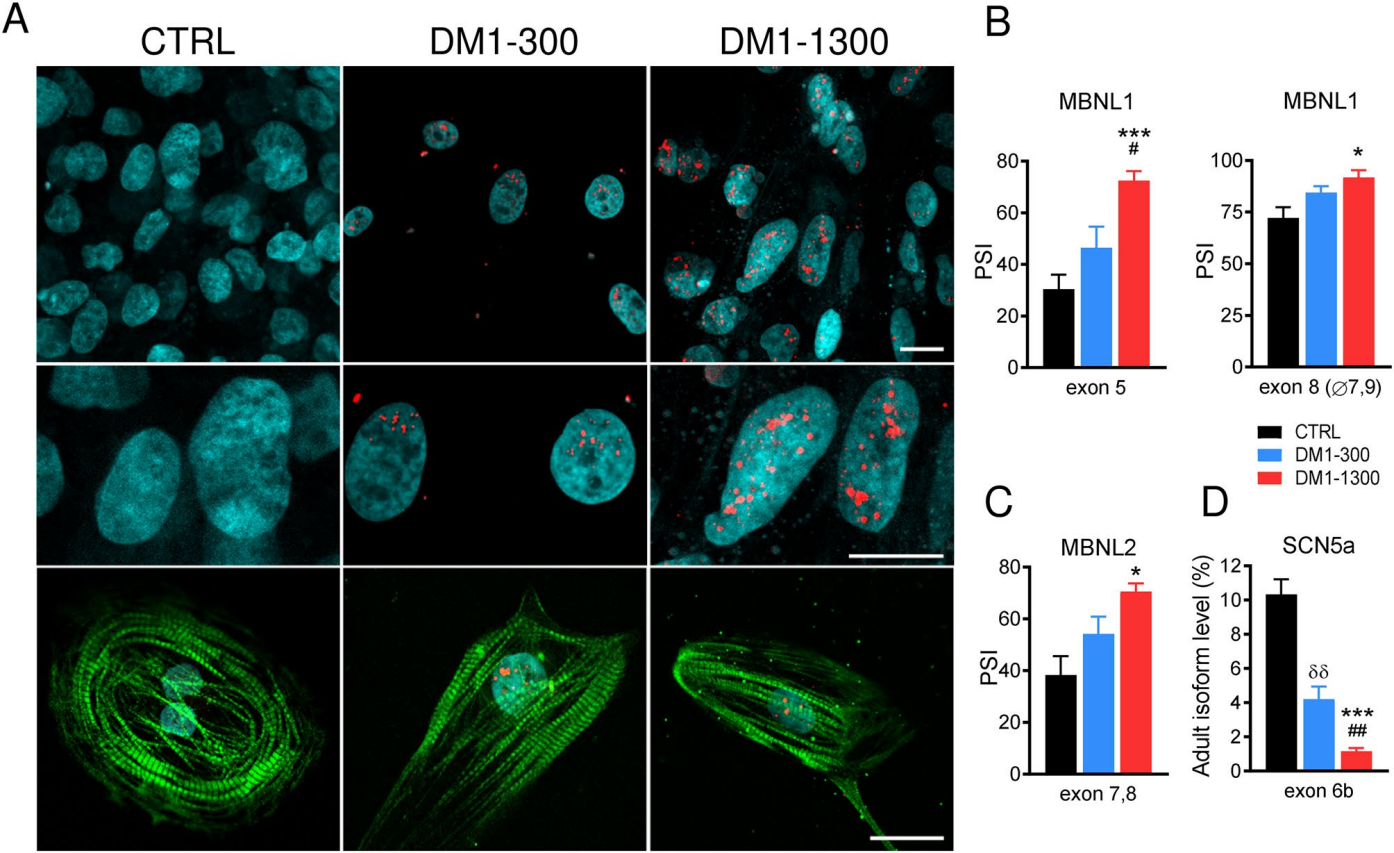
iPSC-CMs from the DM1 patients show hallmarks of the disease. (**A**) Fluorescence in situ hybridization (FISH) using a Cy3-CAG probe (red) to detect RNA foci in CTRL, DM1-300, and DM1-1300 cardiomyocytes. The middle panels show a zoom-in of nuclei, and the bottom panels show cTnT co-expression (green). The nuclei are counterstained with DAPI (cyan). iPSC-CMs from the DM1-300 and DM1-1300 patients have high levels of RNA foci in the nuclei. No foci signals were detected in iPSC-CMs from the CTRL. Scale bar: 20 µM. Images were acquired using Zeiss ImagerM2 LSM confocal microscope and processed with ZEN software (Zeiss). (**B**,**C**) Bar graphs showing splicing mis-regulations of *MBNL1* (**B**) and *MBNL2* (**C**) in iPSC-CMs from the CTRL (n = 3), DM1-300 (n = 3) and DMI-1300 (n = 3). The results are expressed as a percent splice index (PSI) obtained from ASPCR assays. (**D**) RT-qPCR quantification of the percentage of SCN5A mRNA, including adult exon 6b, after 30 days of maturation in iPSC-CMs from CTRL and DM1 iPSC-CMs. Bars indicate SEM. Significant differences were observed between CTRL (n = 3) and DM1-300 (n = 3) (^δδ^p < 0.01), between CTRL and DM1-1300 (n = 3) (*p < 0.05, ***p < 0.001), and between DM1-300 and DM1-1300 (^#^p < 0.05) as determined by ANOVA and Tukey’s post hoc test. A replicate (n) represents an RNA extract from a single independent differentiation.

To determine whether the iPSC-CMs from the two DM1 patients recapitulated the molecular phenotypic hallmark of the disease, we assessed the presence of intranuclear RNA foci together with cardiac troponin T. A Fluorescence in situ hybridization (FISH) analysis revealed multiple foci of aberrant RNA in the nuclei of the DM1-300 and DM1-1300 iPSC-CMs, whereas foci where not observed in the control cells (Fig. 2A). The presence of these nuclear foci is directly linked to splicing regulation defects^20^, or spliceopathies, typical of DM1. To investigate this aspect, we carried out an alternative splicing analysis using endpoint PCR coupled with microcapillary electrophoresis (ASPCR). We interrogated a total of 154 alternative splicing events (ASEs) in selected genes that could be correlated with the cardiac manifestations of the disease (Supplementary Table 1–2). At the end of the validation screens, 16 significantly mis-splicing events were identified in 13 different RNA in the DM1-300 and/or DM1-1300 iPSC-CMs (Fig. S4A,B, Supplementary Table 3). Among them, seven mis-spliced RNA have been identified in previous studies from human DM1 embryo, brain, skeletal muscle or heat tissues (*TTN*^21^, CAST^21^, *PDLIM3*^21,22^, *MAPT*^23^, *ABLIM1*^13,24^, *MYOM1*^25^ and *MBNL1*^26^), three have been identified in previous studies from transgenic mouse models (*APT11A*^27^, *CACNA1D*^28^ and *MBNL2*^29^), one has been identified in iPSC-CM (*ANK3*^30^), and two others that represent, to the best of our knowledge, novel alterations of alternative splicing (*KCNJ12* and *MYH10*). Most of the mis-splicing alterations were detected only in DM1-1300, except for *ATP11A.a*, *CAST.d* and *MYH10.b* isoforms that were overexpressed in both DM1-300 and DM1-1300. *MBNL1* and *MBNL2* have been extensively studied for their role in the pathogenicity of DM1 and different transgenic mice implicating these genes have been generated over the past years. The detection of mis-splicing alterations in their RNA was expected and strengthen our DM1 cell model. Indeed, the expression of the *MBNL1* transcript isoform including exon 5, which regulates protein nuclear localization, and the *MBNL1* transcript isoform including exon 8 (but excluding exons 7 and 9), which regulates protein self-dimerization, were increased in DM1-1300 iPSC-CMs compared to the CTRL iPSC-CMs (Fig. 2B). The transcript isoform of *MBNL2*, including exons 7 and 8, was also increased in DM1-1300 iPSC-CMs compared to CTRL (Fig. 2C).

We next analyzed the splicing profile of *SCN5A* (Na_V_1.5 cardiac sodium channel gene), which has already been reported as being mis-spliced in hearts from DM1 patients^13^. Given the limitations of the ASPCR technique with respect to detecting isoforms of the same size, we performed a standard RT-qPCR using specific primers against neonatal (exon 6a) and adult (exon 6b) isoforms of *SCN5A*. The results revealed a down-regulation of the adult exon 6b isoform in both DM1-300 and DM1-1300 iPSC-CMs compared to CTRL iPSC-CMs (Fig. 2D) at 30 days and 60 days (data not shown) post-differentiation. Since the neonatal and adult isoforms are mutually exclusive, the downregulation of the adult isoform means by default the up-regulation of the neonatal isoform (details in Supplemental Methods).

The level of modification of the splice isoforms pattern found with the ASPCR assay and in *SCN5A* were consistently greater in DM1-1300 than in DM1-300.

### Na_V_1.5 channel gating properties are different in DM1 and control cardiomyocytes

**Figure 3:**
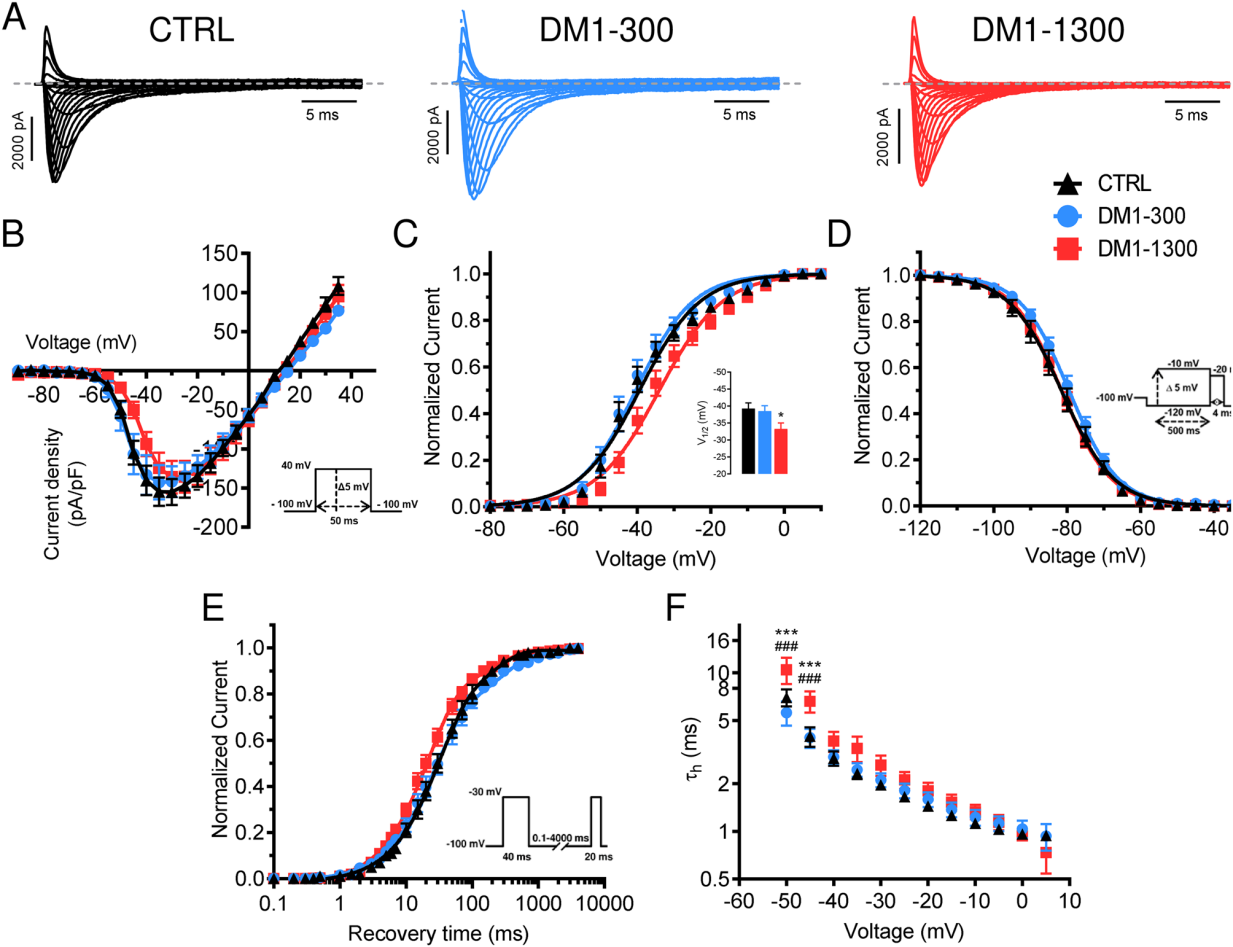
Sodium channels exhibit electrophysiological modifications in patient-specific iPSC-CMs. (**A**) Representative sodium currents recorded in CTRL and DM1 iPSC-CMs. The dashed line represents zero current. (**B**) Normalized current/voltage relationships recorded in CTRL (n = 12), DM1-300 (n = 11), and DM1-1300 (n = 13) iPSC-CMs. Sodium current densities were measured by normalizing current amplitudes to membrane capacitance and were plotted against voltage. (**C**) Steady-state activation of sodium currents from CTRL and DM1 iPSC-CMs. Activation curves were generated using a standard Boltzmann distribution: G(V)/G_max_ = 1/(1 + exp(- (V-V_1/2_)/k)). Inset shows graph of V_1/2_. (**D**) Steady state inactivation of CTRL (n = 12) DM1-300 (n = 9) and DM1-1300 (n = 12). Inactivation currents were obtained using 10-ms test pulses to – 10 mV after a 500-ms pre-pulse to potentials ranging from – 140 to – 20 mV. The inactivation values were fitted to a standard Boltzmann equation: I(V)/I_max_ = 1/(1 + exp((V − V_1/2_)/k)) + C. (**E**) Recovery from inactivation values recorded from CTRL (n = 9), DM1-300 (n = 9), and DM1-1300 (n = 9) iPSC-CMs. The cells were depolarized to – 30 mV for 40 ms from a holding potential of – 100 mV to inactivate the Na^+^ channels. Test pulses were then applied to – 30 mV for 20 ms to measure current amplitudes, with an interval ranging from 0.1 to 4000 ms. The resulting curves were fitted with a two-exponential equation: (A_fast_(1 – exp(− t/τ_fast_)) + Aslow(1 − exp(− t/τ_slow_)) + C). (**F**) The time constants of fast inactivation decay were plotted as a function of voltage for the CTRL and DM1 iPSC-CMs. The time constants were obtained using a single exponential function: (A(exp(− t/τ) + C). Bars indicate SEM. *p < 0.05, ***p < 0.001 (CTRL vs DM1-1300) and ^###^p < 0.05 (DM1-300 vs DM1-1300) by ANOVA and Tukey’s post hoc test.

In cardiomyocytes, the sodium current, I_Na,_ initiates and causes the rise of action potentials (APs). Cardiac Na_V_1.5 channels are by far the main contributor to I_Na_. To determine whether I_Na_ is affected in DM1 iPSC-CMs, we performed voltage-clamp analyses in disaggregated single cells. The experiments were done at day 30 post-differentiation since we did not find out any significant evolution in the splicing profile of SCN5a between groups with prolonged culture time. iPSC-CMs from DM1-300, DM1-1300, and CTRL produced robust I_Na_ densities (142.8 ± 19.7 pA/pF, 136.0 ± 20.7 pA/pF, and 155.0 ± 16.7 pA/pF, respectively) (Fig. 3A,B). However, the I_Na_ current–voltage (I/V) relationship for the DM1-1300 iPSC-CMs revealed a small shift toward more depolarized voltages (Fig. 3B). To further study the I_Na_ activation, the I/V curves were converted to conductance and were plotted against voltage (G/V). The curves were then fitted using a Boltzmann function (Fig. 3C). The results revealed that the V_1/2_ of the voltage-dependence of I_Na_ activation of DM1-1300 was significantly more positive (CTRL: V_1/2_ = – 39.2 ± 1.7 mV; DM1-300: V_1/2_ = – 38.5 ± 1.6 mV; DM1-1300: V_1/2_ = – 33.3 ± 1.8 mV), while the slopes of the curves were similar for all conditions (CTRL: k_v_ = 7.4 ± 0.6; DM1-300: k_v_ = 6.7 ± 0.4; DM1-1300: k_v_ = 7.3 ± 0.5). Several milliseconds following their activation, sodium channels switch to a non-conducting state in a process called fast inactivation, which contributes to repolarization of APs. The steady-state inactivation was then analyzed. The results showed that there is no significant shift in the curves between the DM1-300, DM1-1300, and CRTL iPSC-CMs (CTRL: V_1/2_ = – 81.7 ± 1.6 mV; DM1-300: V_1/2_ = – 78.0 ± 1.0 mV, DM1-1300: V_1/2_ = − 81.5 ± 1.0 mV; Fig. 3D). The voltage-dependence of recovery from inactivation was also assessed. There were no significant differences between the resulting time constants of recovery between the 3 groups (Fig. 3E). However, DM1-1300 hiPSC-CMs exhibited slower inactivation kinetics at hyperpolarized voltages than the DM1-300 and CTRL iPSC-CMs as calculated by fitting the current decays with exponential functions (Fig. 3F).

### DM1-1300 cardiomyocytes exhibit increased calcium currents

**Figure 4:**
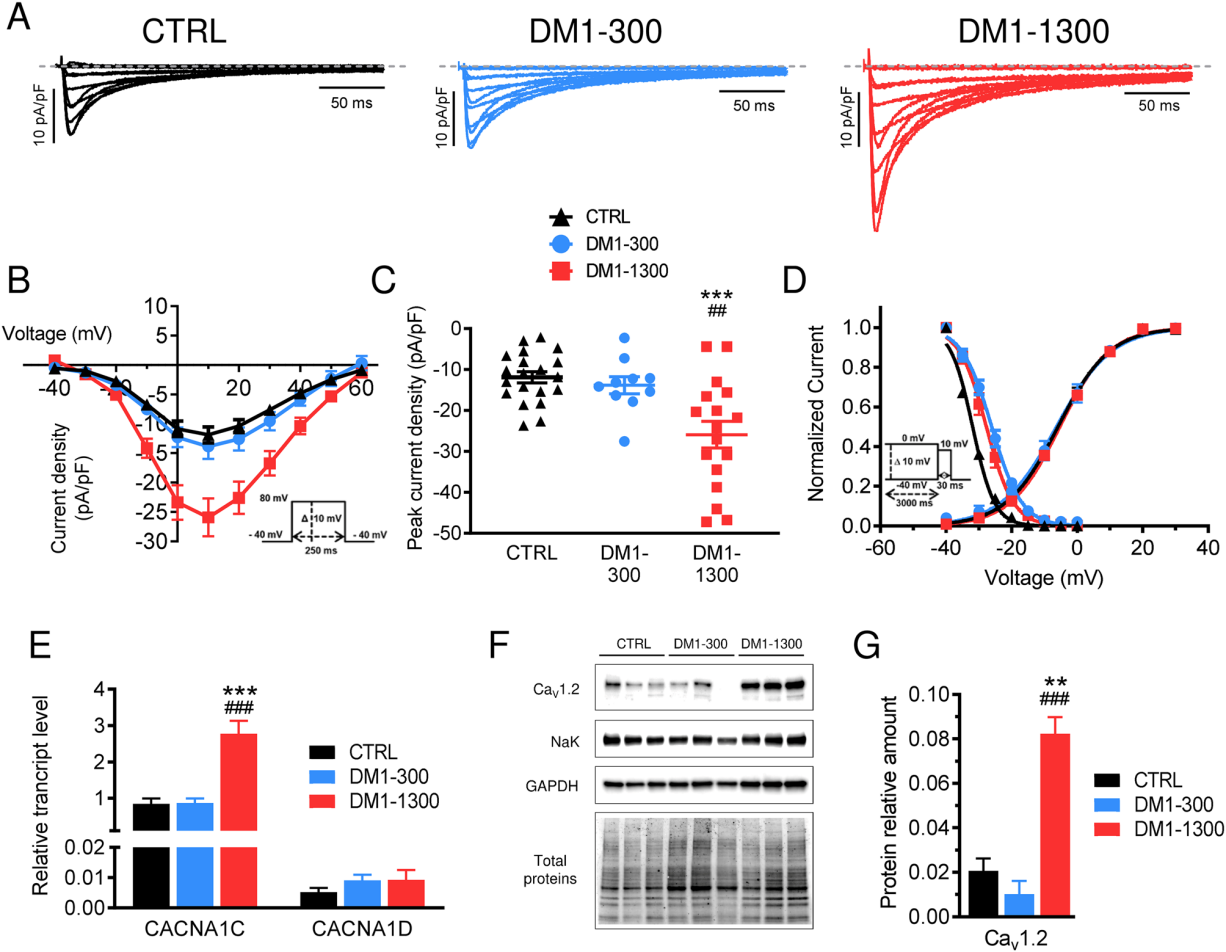
L-type calcium current density is higher in iPSC-CMs from the DM1-1300 patient. (**A**) Representative L-type Ca^2+^ current traces recorded from CTRL, DM1-300, and DM1-1300 iPSC-CMs. The dashed line represents zero current (**B**) L-type Ca^2+^ channel current–voltage relationships recorded in CTRL (n = 20), DM1-300 (n = 10), and DM1-1300 (n = 17) iPSC-CMs. The current was normalized to the capacitance (pF) of the cells. (**C**) Dot plot showing the L-type Ca^2+^ channel current densities recorded at 10 mV. (**D**) Voltage-dependence of steady-state activation (n = 10–20) and inactivation (n = 7–9) of L-type channel Ca^2+^ currents. (**E**) RT-qPCR quantification of the expression of *Ca*_*V*_*1.2* mRNA in iPSC-CMs. Results are expressed relative to the expression of *GATA4* mRNA. (**F**) Western blot for the quantification of Ca_V_1.2 protein level in CTRL, DM1-300 and DM1-1300 iPSC-CMs. The membrane was cut in three sections and blot with the selected antibody. Panel 1 to 4 (from top to bottom) show Ca_V_1.2, Na/K ATPase pump, GAPDH and total proteins (uncut blot, stain-free technology) signals, respectively. For each group, three samples from three independent differentiation were analyzed. The four panels came from the same gel/blot. Brightness and contrast were adjusted. No nonlinear adjustments were applied. (**G**) Quantification of Ca_V_1.2 protein level in CTRL (n = 3), DM1-300 (n = 3) and DM1-1300 (n = 3) from western blot is shown in panel F. Results are expressed relative to total protein signal. Bars indicate SEM. **p < 0.01, ***p < 0.001 (CTRL vs DM1-1300) and ^##^p < 0.01, ^###^p < 0.001 (DM1-300 vs DM1-1300) as determined by ANOVA and Turkey’s post hoc test.

L-type calcium channels are responsible for excitation–contraction coupling in cardiac muscle by conducting the inward Ca^2+^ current (I_CaL_) that triggers Ca^2+^ release from the sarcoplasmic reticulum^31^. We measured this I_CaL_ in the DM1 and CTRL iPSC-CMs (Fig. 4A). Interestingly, I_CaL_ recorded from DM1-1300 iPSC-CMs were characterized by a two-fold increase in I_CaL_ density (Fig. 4B,C). The inactivation of I_CaL_ was also affected, where a significant shift of the steady-state inactivation curves of 8 mV and 6 mV to depolarized potentials was observed in DM1-300 and DM1-1300 iPSC-CMs, respectively, compared to CTRL hiPSC-CMs (CTRL: V_1/2_ = 34.7 ± 0.4 mV; DM1-300: V_1/2_ = – 26.1 ± 0.7 mV; DM1-1300: V_1/2_ = – 29.0 ± 1.2 mV) (Fig. 4D). However, the activation of these Ca^2+^ channels was the same for the DM1 and CTRL hiPSC-CMs, as revealed by the G/V curves (CTRL: V_1/2_ = – 4.6 ± 1.0 mV; DM1-300; V_1/2_ = – 5.3 ± 1.6 mV; DM1-1300: V_1/2_ = – 4.2 ± 1.1 mV (Fig. 4D).

We next measured the level of transcripts of the two main L-type Ca^2+^ channels expressed in cardiac muscle, *CACNA1C* and *CACNA1D*, which encode for the Ca_V_1.2 and Ca_V_1.3 channels, respectively. The qPCR data revealed that *CACNA1C* transcripts are significantly upregulated in the DM1-1300 hiPSC-CMs compared to the DM1-300 and CTRL hiPSC-CMs, which is consistent with the observed increase in current density (Fig. 4E). The levels of *CACNA1D* transcripts were not only similar between the three experimental groups but were also much lower than the levels of *CACNA1C* transcripts. The upregulation of *CACNA1C* was confirmed by western blot, where a 4- and eightfold increase in Ca_V_1.2 protein levels was seen in iPSC-CMs from DM1-1300 compared to control and DM1-300, respectively (Fig. 4F,G).

### DM1 cardiomyocytes have altered action potentials

**Figure 5:**
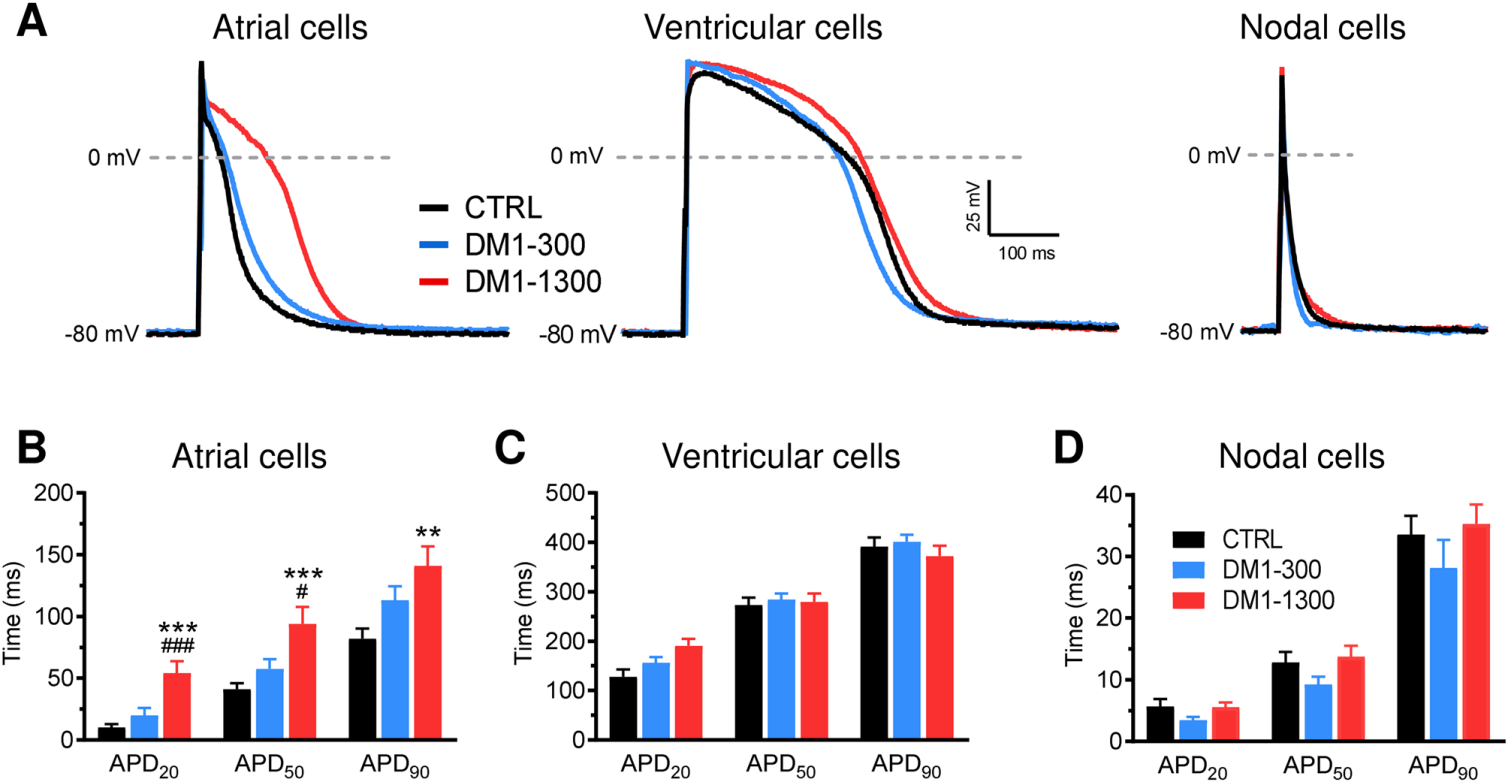
APD prolongation revealed by patient-specific iPSC-CMs. (**A**) Representative AP traces of atrial, ventricular, and nodal-like cells recorded in CTRL, DM1-300, and DM1-1300 iPSC-CMs. (**B**) Bar graph summarizing the APD at 20%, 50%, or 90% of repolarization in atrial-like (n = 19–21), (**C**) ventricular-like (n = 12–25), and (**D**) nodal like (n = 14–21) cells at a stimulation frequency of 1 Hz. Bars indicate SEM. **p < 0.01, ***p < 0.001 (CTRL vs DM1-1300) and ^#^p < 0.05, ^###^p < 0.001 (DM1-300 vs DM1-1300) as determined by ANOVA and Turkey’s post hoc test.

**Figure 6:**
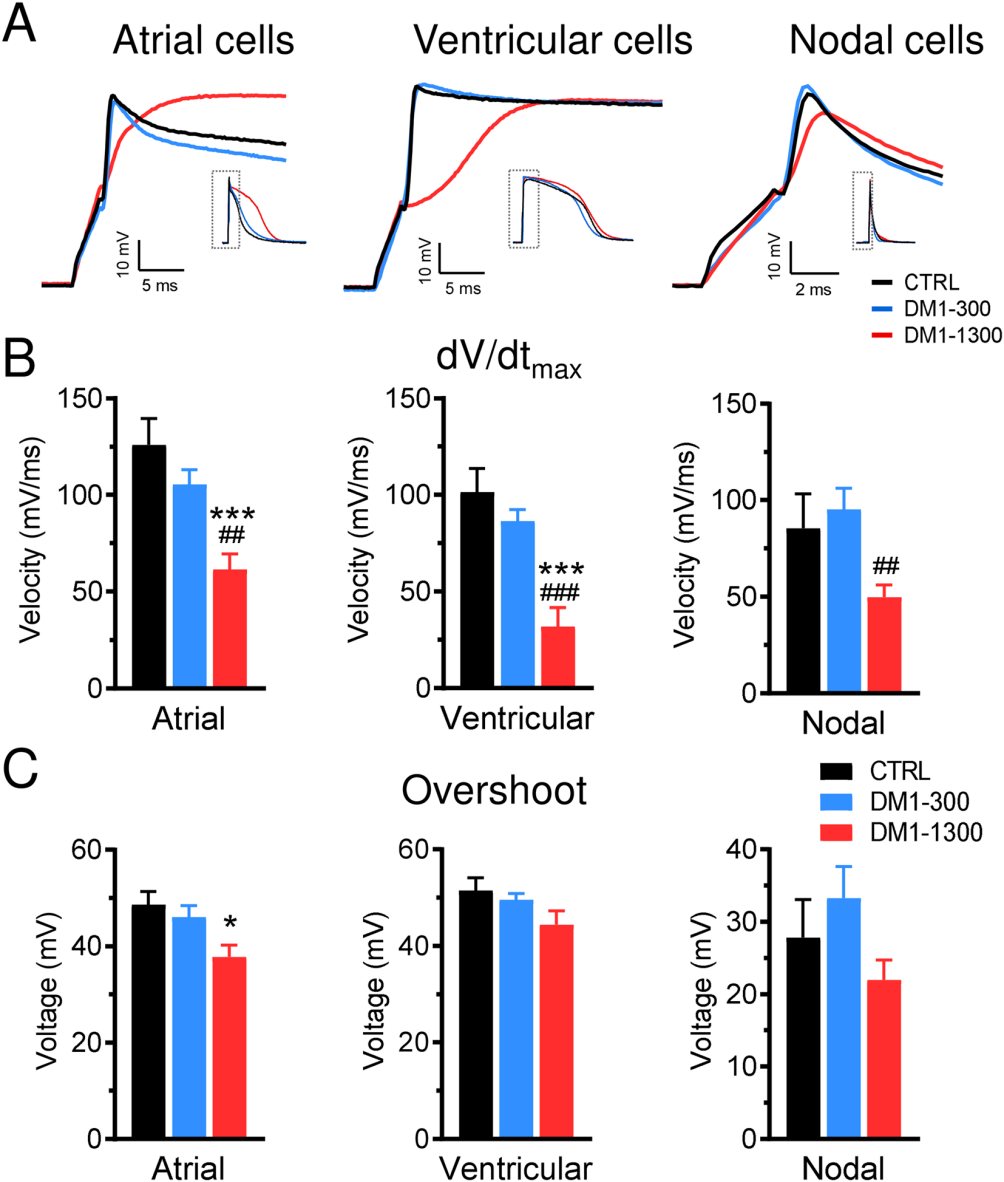
Action potentials dV/dt and overshoot are affected in DM1 iPSC-CMs. (**A**) Close-up view on the rising phase of representative APs recorded at a stimulation frequency of 1 Hz. (**B**) Bar graphs showing the maximal upstroke velocity (dV/dt_max_) and overshoot (**C**) of APs recorded in atrial-like (n = 21–23), ventricular-like (n = 12–25), and nodal-like (n = 14–21) cells at a stimulation frequency of 1 Hz. Bars indicate SEM. *p < 0.05, ***p < 0.001 (CTRL vs DM1-1300) and ^##^p < 0.01, ^###^p < 0.001 (DM1-300 vs DM1-1300) as determined by ANOVA and Turkey’s post hoc test.

To gain insights into the mechanism leading to DM1 cardiac arrhythmogenesis, APs were recorded in single iPSC-CMs. As expected, APs from CTRL, DM1-300, and DM1-1300 hiPSC-CMs exhibited diverse shapes that can be attributed to different cardiomyocyte cell types, that is, ventricular, atrial, and nodal cells (Fig. 5A). AP parameters were analyzed, taking these subpopulations into account (details in Supplementary Method). Although hiPSC-CMs generate spontaneous APs, the experiments were performed on cardiomyocytes stimulated at different frequencies to normalize the beating rate. AP recorded in DM1-1300 hiPSC-CMs exhibited a significant increase in their duration (APD) as measured at 20, 50, and 90% of repolarization in atrial-like cells compared to DM1-300 and CTRL (Fig. 5A,B and Fig. S5A). For example, APD_50_ in DM1-1300 was increase by 37 ms and 53 ms compared to DM1-300 and CTRL, respectively. APD were found similar in ventricular-like and nodal-like cells between all groups (Fig. 5C,D and Fig. S5B,C). AP recorded in DM1-1300 also revealed a marked decrease in their upstroke velocity (dV/dt) compared to DM1-300 and CTRL (Fig. 6A,B and Fig. S6). The dV/dt reduction was observed in all hiPSC-CMs subpopulations recorded. Concerning, the AP overshoot, a slight reduction was observed in DM1-1300 but only significant in atrial-like cells (Fig. 6C and Fig. S6B). Finally, no difference was observed in the resting membrane potential between all groups (Fig. S7). It is also important to note that no significative alteration in APs parameters was seen between hiPSC-CM from DM1-300 and CTRL.

### DM1-1300 hiPSC-CMs have a slower conduction velocity

**Figure 7:**
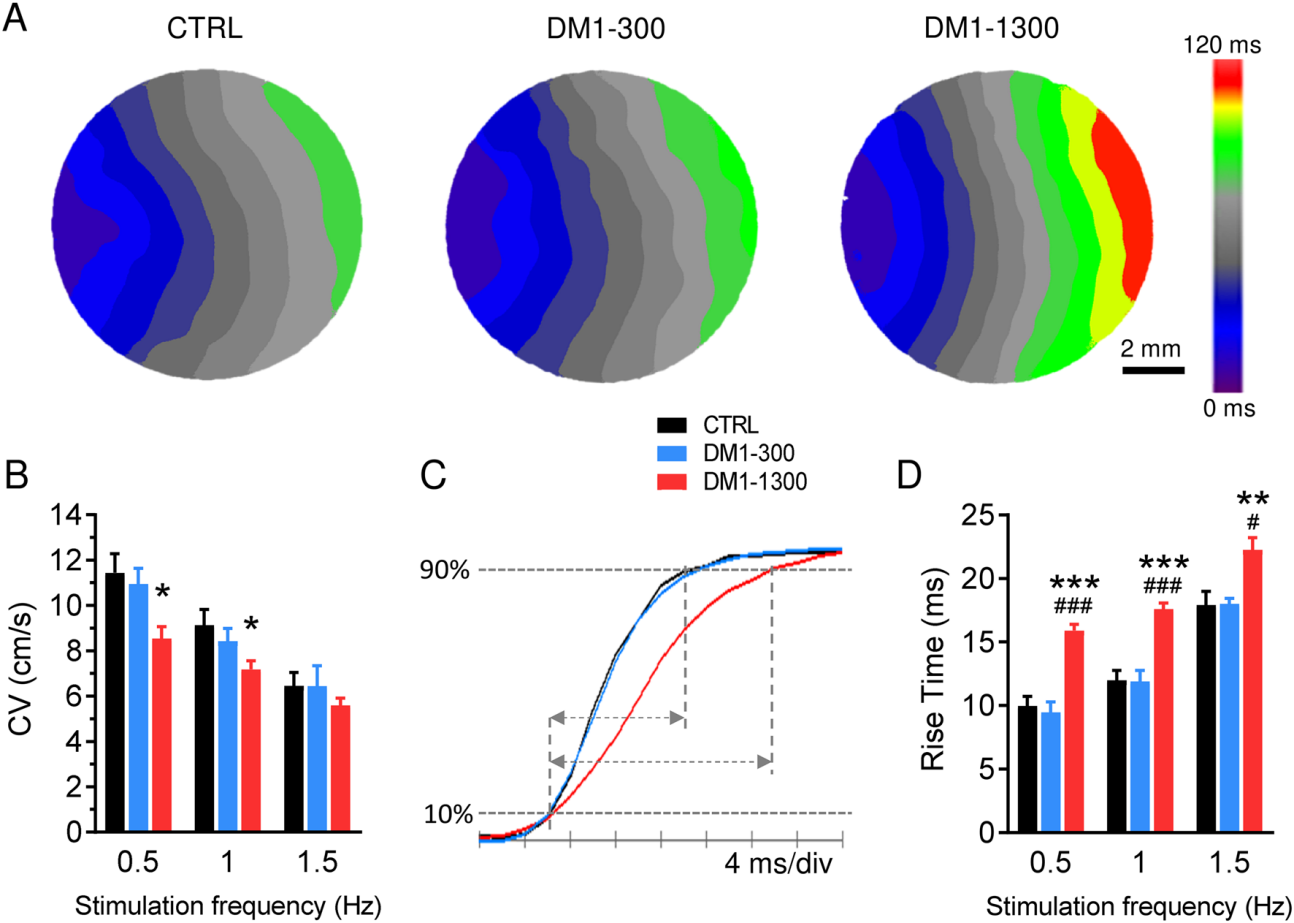
Conduction velocity is slower in DM1-1300 iPSC-CMs. (**A**) Representative activation maps at a pacing of 1 Hz in iPSC-CM from CTRL, DM1-300 and DM1-1300. (**B**) Bar graph summarizing the CVs measured at stimulation frequencies of 0.5, 1 and 1.5 Hz in CTRL (n = 22–23), DM1-300 (n = 9–19) and DM1-1300 (n = 13–22). (**C**) Representative optical AP showing the rise segment of APs. The rise time was calculated between 10 and 90% of the rise course as indicated by the arrows. (**D**) Histograms summarizing the rise time of APs measured at stimulation frequencies of 0.5, 1 and 1.5 Hz in CTRL (n = 22–23), DM1-300 (n = 9–17) and DM1-1300 (n = 13–22). Bars indicate SEM. *p < 0.05, **p < 0.01, ***p < 0.001 (CTRL vs DM1-1300) and ^#^p < 0.05 and ^###^p < 0.001 (DM1-300 vs DM1-1300) as determined by ANOVA and Turkey’s post hoc test. A replicate (n) represents a single coverslip of iPSC-CMs on which the optical mapping was performed.

hiPSC-CMs monolayers are a powerful tool for the study of fundamental mechanisms that underlie the electrophysiological abnormalities at the tissue level. Using a voltage-sensitive dye and optical mapping methods, we next evaluated the conduction of electrical impulses throughout iPSC-CMs monolayers. Upon stimulation at 0.5 and 1 Hz, the conduction velocity (CV) measured throughout the monolayers was significantly reduced in DM1-1300 (8.5 ± 0.5 cm/s at 0.5 Hz and 7.2 ± 0.4 cm/s at 1 Hz) compared to CTRL (11.4 ± 0.8 cm/s at 0.5 Hz and 9.1 ± 0.7 cm/s at 1 Hz) (Fig. 7A,B and Supplementary Video 1 and 2). No CV reduction was observed between DM1-300 and CTRL hiPSC-CM monolayers. In Fig. 7A, a representative example of activation maps obtained by pacing at 1 Hz show the increase in duration of the propagation of electrical signal in DM1-1300 iPSC-CMs. The rise time of voltage APs was also affected in the iPSC-CM monolayers where it was prolonged in DMI-1300 (Fig. 7C,D).

## Discussion

We conducted a molecular and electrophysiological analysis using cardiomyocytes derived from iPS cells (iPSC-CMs) of two individuals with DM1. We first showed that the nuclei of iPSC-CMs from the DM1 patients with two different CTG triplet repeat lengths accumulated toxic foci containing CUG repeats, the molecular hallmark of DM1. These foci are the result of the accumulation of mutant elongated *DMPK* RNA transcripts and the sequestration of the RNA-binding proteins MBNL, and as such constitute the starting point of the spliceopathy phenomenon. To determine the involvement of spliceopathies in cardiac malfunctions, we carried out a mis-splicing ASPCR analysis on iPSC-CM from the two DM1 patients (DM1-300 versus DM1-1300). We identified 16 mis-splicing transcripts in iPSC-CMs from the two DM1 patients. As expected, *MBNL1* and *MBNL2* were mis-spliced in DM1 iPSC-CMs. The overexpression of *MBNL1* transcripts containing exon 5 found in the present study has also been reported in biopsies from DM1 skeletal muscle and more recently from DM1 iPSC-CMs^26,32,33^. *MBNL2* was also found to be mis-spliced in previous studies. As reported here, the *MBNL2* transcript containing exon 7 is also overexpressed in a DM1 mouse model, while the transcript containing exon 5 is overexpressed in iPSC-CMs from other DM1 patients^29,32^. In the present study, ASPCR did not cover this locus. We cannot thus reach any conclusions regarding the expression of *MBNL2*/exon 5. As mentioned in the results section, several mis-spliced RNA found in our ASPCR assay have also been identified in previous studies. We found remarkable mis-splicing events in RNAs that code proteins directly implicated in cardiac electrical function such as: *ANK3* coding for ankyrin-3 protein which interacts with Na_V_1.5 channels to regulate its membrane localization, *CACNA1D* coding for the L-type calcium channel Ca_V_1.3 involved in automaticity of cardiomyocytes, or *KCNJ16* coding for the potassium inwardly-rectifying channel K_ir_5.1 involved in homeostasis of membrane potential. We also found mis-splicing events in RNAs that code for proteins involved in the mechanical function of cardiomyocytes such as: *PDLIM3* and *ABLIM3* coding for two different actin/actinin-binding LIM proteins present in the sarcomeric Z-disks structure^34,35^, or *MYOM1* and *TTN* coding respectively for myomesin-1 and titin proteins which compose the M-line of the sarcomere and involved in hypertrophic cardiomyopathy^36^. Detected by RT-qPCR instead of ASPCR assay, the splicing switch from the adult to the neonatal isoforms in *SCN5A* mRNAs are one of particular interest. Previous studies have also reported that the *SCN5A* neonatal isoform is overexpressed in DM1 heart samples, including samples from humans^13,37^.In terms of *SCN5A*, which codes for Na_V_1.5, the cardiac Na^+^ channel, the increase in the neonatal isoform was consistent with the gating changes observed in I_Na_ in DM1-1300 cardiomyocytes. Indeed, the reduced excitability of Na_V_1.5 channels recorded in DM1-1300 cardiomyocytes (shift of activation to depolarized potentials) was similar to the shift already observed between the neonatal and adult isoforms^13,38^. We are attempted to speculate that the gating changes seen in DM1-1300 could be related to the increased expression of the neonatal splice variant. However, we cannot exclude that other factors can contribute to this shift. Because the critical roles that Na_V_1.5 plays in the initiation and rise of cardiac APs, a reduction in the excitability of Na_V_1.5 channels will have a direct impact on the electrical conduction velocity of cardiac myocytes. This is exactly what we observed with patient-specific iPSC-CMs, where the overshoot was slightly reduced in atrial cells and the upstroke velocity (dV/dt) was markedly reduced in all cell subpopulations, which may lead to conduction delay and predisposition to cardiac arrhythmias. These change in APs profiles were also correlated with the number of CTG repeats and the severity of the patients’ cardiac phenotype. The forced expression of the neonatal isoform into adult mouse heart was evaluated in two previous studies and succeeded in reproducing the conduction defects and cardiac arrhythmias seen in DM1^13,39^. The ECG parameters from individuals with cardiac-conduction abnormalities caused by loss-of-function mutations in Na_V_1.5 also share some similarities with those of DM1 patients, including prolongation of the PR interval and of the QRS duration^37,40^. The inactivation kinetics was another Na_V_1.5 biophysical parameters that appears impacted in DM1-1300, which showed slower kinetics but only at hyperpolarized voltages. This is probably a by-product of the shift of activation and not per se a modification in the inactivation process of the channels. In the light of these results, we believe that the switch from the adult to the neonatal isoform of Na_V_1.5, could be a key molecular contributor in the development of cardiac electrical manifestations in DM1.

However, the alteration of the I_Na_ alone does not explain the full pattern of cardiac conduction. The overexpression of Ca_V_1.2 at transcripts and protein levels in DM1-1300 hiPSC-CMs is in accordance with the increased I_CaL_ density and may also contribute to these electrical abnormalities. In cardiomyocytes, Ca_V_1.2 channels affect the duration of the action potential by mediating the plateau phase. The increase in I_CaL_ density together with its shift of the inactivation curve to depolarized potentials are both gain-of-functions of Ca_V_1.2 in DM1-1300 hiPSC-CMs that contribute to and could explain the APD prolongation. Clinical evidence also supports the link between the alteration of Ca_V_1.2 function and cardiac diseases. Indeed, in Timothy syndrome, gain-of-function mutations in Ca_V_1.2 increase the influx of Ca^2+^ into the cells and prolong the APD, resulting in severe cardiac repolarization abnormalities^41^. The prolongation of APD seen in DM1-1300 could thus be an additional factor that increases the risk of arrhythmias in DM1. Furthermore, the large I_CaL_ observed in iPSC-CMs from the DM1-1300 patient may increase intracellular Ca^2+^ levels, leading to a decrease in the conductance of gap junctions and thus conduction delays. Indeed, acute increases in intracellular Ca^2+^ levels are associated with gap junctional uncoupling and decreased conduction^42^. Similar I_CaL_ increases have been reported in the skeletal muscles from DM1 patients^43^. Our results are also consistent with a previous study that reported that Ca_V_1.2 protein was overexpressed in heart samples from DM1 patients with cardiac conduction defects and that the overexpression was caused by the misregulation of miR-1 due to MBNL1 nuclear sequestration^44^. More recently, a Ca_V_1.2 subunit, *CACNA2D3*, known to positively regulate the channel’s cell surface abundance, was found upregulated in a *Drosophila* DM1 model exhibiting cardiac conduction defects. The overexpression alone of this subunit in the *Drosophila* was sufficient to reproduce asynchronous heartbeats. In addition, this Ca_V_1.2 subunit was also found elevated in hearts from DM1 patient with conduction defects^45^. These results support ours and suggest a predominant role of calcium channels in cardiac electrical abnormalities in DM1 patients.

Nonetheless, our results are not in line with those of Spitalieri et al. demonstrating a reduction in the Ca_V_1.2 transcript in iPSC-CMs from DM1 patients^32^. However, their work was conducted on cells from DM1 patients with normal EKG and free of cardiac symptoms, while one of our patients had major EKG abnormalities that required an implantation of a pacemaker. In fact, we did not find any Ca_V_1.2 current or transcript/protein increase in our cells from the cardiac asymptomatic patient (DM1-300). Furthermore, in Spitalieri et al. study, unlike us, the authors found prolongation of the APD in their ventricular cells (atrial cells data was not provided). This discrepancy could be explained by a different experimental approach. While their AP recording was carried out in non-stimulated spontaneously beating cells which may have had different frequencies of firing, we analyzed stimulated AP from cells maintained at a holding potential of − 80 mV. This approach allowed us to control the firing frequencies to normalize the AP parameters from cell to cell.

However, we cannot exclude the possible role of other ion channels than calcium channels in the prolongation of APD. Recently, a transgenic mouse model (LC15) with high expression of expanded CUG-repeat RNA in the heart also exhibited prolonged APD in cardiomyocytes but related at least in part to a reduction in I_to,slow_ (transient outward potassium current) density^46^. These are very interesting results, but further warrant the assessment of the I_to,slow_ in the prolonged APD seen in DM1-1300. This highlights the complexity of the contributing factors that can influence the duration of the action potential. While the upstroke velocity (mainly influenced by Na_V_1.5 channels) was altered in all iPSC-CM subpopulations, APD prolongation was observed only in atrial cells in our iSPC-CMs. The lack of effect on ventricular (and nodal) APD cells could be explained by possible compensating effects from other ion channels that were not studied here.

Patient 1 with 1300 CTG triplet repeats had a pacemaker because of conduction abnormalities. Optical mapping experiments reproduce the conduction defects by demonstrating that DM1-1300 iPSC-CMs have delayed conduction in hiPSC-CMs monolayers compared to CTRL. This is the first time that conduction delays were recorded and reported in a human DM1 cellular model. A previous study also showed a reduction in CVs but in a MBNL knock-out mice model^47^. Considering that the conduction velocity depends on the rate of tissue depolarization, which is related to the slope of phase 0 of the AP, the reduction of dV/dt observed in APs from DM1-1300 possibly explain in part this delayed conduction.

It is noteworthy that no significant electrical changes (ionic currents and optical mapping study) were observed between CTRL and DM1-300. This is evocative of the clinical condition of the patient, who has no manifestation of cardiac manifestations. The low level of mis-splicing events found in DM1-300 could be an explanation for this phenotype/clinical condition. Indeed, in the ASPCR assay, only three events of splicing alterations were detected in DM1-300 compared to the 16 events found in DM1-1300. Not to mention the lower level of mis-spliced *SCN5A* in DM1-300 compared to DM1-1300. Besides, an association between the level of mis-splicing event in subjects with DM1 and the severity of the disease has been demonstrated. For example, Thornton’ group revealed a correlation between the level of *MBNL1* exon 5 inclusion (as seen in DM1-1300) and muscle weakness in DM1 subjects^33^. In addition, it has been shown that MBNL1 protein directly regulate the switch between *SCN5A* neonatal and adult isoforms in hearts^13^. However, the hypothesis that link the level of mis-splicing events, and especially *SCN5A* mis-splicing level, with the electrical dysfunction into cardiomyocytes need to be validated with more iPSC cell lines from several DMI subjects.

In conclusion, by using hiPSC-CM, we succeed to develop a DM1 disease model and highlighted two major perturbations in ion channels in DM1, one affecting Na^+^ channels and the other affecting Ca^2+^ channels. This work sheds light on the cardiac manifestations of DM1 and provide a better understanding of the mechanism underlying the electrical cardiac alterations observed in DM1 patients. A more detailed understanding of this mechanism may lead to the development of better treatments strategies for DM1. For example, by monitoring the conductions velocities and ionic currents in iPSC-CM from DMI patients, we can validate the efficiency of new therapeutics, such as antisense oligonucleotides (AOs), to conduct target biologic screenings to find agents that normalize the cardiac electrical abnormalities. In the future, this approach can ultimately lead to personalized medicine.

### Study limitations

This study reveals new insight into cardiac electrical dysfunction in DM1 with some limitations: given the complexity of the disease and the broad range of clinical symptoms, the study of a larger cohort of DM1 patients with different CTG repeat would be beneficial for both (1) confirming our discovery about ion channels and conductions velocity abnormalities, and (2) strengthening the correlation between the level of mis-spliced RNAs and cardiac electrical defects. In addition, hiPSC-CMs are known to a have a phenotype of the neonatal rather than the adult. This aspect can impact on our study results because the level of *SCN5a* adult isoform is constitutively underexpress in all hiPSC-CM cell lines. Thereby, the decrease of adult isoform seen in DM1-300 and DM1-1300 is probably undervalued. The study focused on the role of Na^+^ and Ca^2+^ channels, but we cannot exclude that other ion channels or contractile proteins may be involved as previously discussed. Further studies are warranted to assess the role of these potential additional factors in DM1’s cardiac dysfunction.

## Supplementary Information

Supplementary Video 1.

Supplementary Video 2.

Supplementary Information.

Supplementary Table 1.

Supplementary Table 2.

Supplementary Table 3.
