# Supplementary material for: iPSC-derived cardiomyocytes from patients with myotonic dystrophy type 1 have abnormal ion channel functions and slower conduction velocities
Source: Sci Rep. 2021 Jan 28;11:2500. doi: 10.1038/s41598-021-82007-8 (PMC7844414; doi:10.1038/s41598-021-82007-8)
Supplement: Supplementary file 3 — Supplementary Information. [file 41598_2021_82007_MOESM3_ESM.docx]

**iPSC-derived cardiomyocytes from patients with myotonic dystrophy type 1 have abnormal ion channel functions and slower conduction velocities**

Hugo Poulin^1^, Aurélie Mercier^1^, Mohammed Djemai^1^, Valérie Pouliot^1^, Isabelle Deschenes^2^, Mohamed Boutjdir^3,4,5^, Jack Puymirat^6^, and *Mohamed Chahine^1,7^

**Supplementary Information**

**Supplementary Methods:**

**Production, derivation, culture and characterization of patient-specific hiPSCs**

Skin biopsies from a healthy donor and two DM1 patients were collected with informed consent and in accordance with the guidelines of the Centre de Recherche du Centre Hospitalier Universitaire de Québec. The dermal fibroblasts were reprogrammed and characterized at the LOEX core facility (Quebec City, Canada) using the OCT3/4, SOX2, KLF4, and C-MYC factors and the non-integrating Sendai virus method. Three iPS clonal lines from each individual were generated, expanded, and characterized. The characterization consisted of evaluating the length and stability of the CTG repeat by Southern blot, the expression of pluripotency-associated proteins, the gene expression of pluripotency markers, the ability to differentiate into germ layers, the identity of the original cells with the hiPSCs, and the karyotype. The expression of surface markers (SSEA-4 and Tra-1-60) and an intracellular marker (OCT4) was evaluated using flow cytometry and immunofluorescence. Pluripotency markers (OCT4, NANOG, SSEA4, TRA-1-60, TRA-1-81) were evaluated by immunofluorescence and by RT-qPCR for endogenous OCT4, Nanog, endogenous Sox2, REXT, Dnmt3b, and hTERT. The ability to differentiate into germ layers was evaluated by the formation of embryonic bodies followed by RT-qPCR to assess AFP, SOX17, and GATA4 (endoderm markers), MSX1, KDR, and GATA2 (mesoderm markers), and PAX6, TUBB3, and NCAM (ectoderm markers) levels. The identity of the hiPSC cells with the original cells was verified using a short tandem repeat (STR) analysis. Karyotyping was performed to rule out possible chromosomal abnormalities (>3-10 Mb). A Southern blot analysis of the 3’-CTG expansion in the DMPK gene in an iPSC culture from the DM1-1300 patient was performed to determine whether the expansion remained stable over time in culture.

**Patient-specific hiPSC cardiomyocyte differentiation**

Three iPSC cell lines, one from each individual, were used to perform all the experiments. iPSCs were grown on hESC-qualified Matrigel in mTeSR1 media (StemCell Technologies, Vancouver, BC, Canada) and were routinely passaged using dispase. The cells were exposed to a series of reagents in a time–controlled manner to induce differentiation via the Wnt signaling pathway^1,2^. Briefly, 5 days prior to the initiation of differentiation, hiPSC colonies were dissociated into single cells using TrypLE express. iPSCs were seeded on hESC-qualified Matrigel and were cultured in mTeSR1 medium, which was changed daily. On day 0, differentiation was initiated using RMPI 1640 medium supplemented with B27 supplement minus insulin (RPMI/B27-insulin) and 6 μmol/L of CHIR99021 (LC Laboratories, Woburn, MA, USA). On day 2, the medium was replaced with RPMI/B27-insulin. On day 3, the medium was replaced with RPMI/B27-insulin and 2 μmol/L of Wnt-C59 (LC Laboratories). On days 5 and 7, the medium was replaced with RPMI/B27-insulin. From day 9 onwards, the medium was replaced every two days with RMPI 1640 supplemented with B27 supplement. Beating cells appeared between days 7 and 12. Based on recently published reports, this process results in highly efficient iPSC differentiation into cardiac myocytes, with more than 80% beating cells.

**ASPCR analyses**

Alternative splicing analyses by end-point RT-PCR (ASPCR) were carried out at the Université de Sherbrooke RNomics Platform, Sherbrooke, Canada. Briefly, total RNA was extracted from iPSC-CMs on day 30 of differentiation using Trizol (Life Technologies, Carlsbad, CA, USA). Three RNA samples from each group (CTRL, DM1-300, and DM1-1300) from three independent differentiations were pooled. cDNA from these pools was synthesized, and a first screen of 154 alternative splicing events in 54 genes that are susceptible to changes was performed. A second screen using three individual samples from each iPSC cell line was then performed to confirm the hits from the first screen. In each screen, the amplified products were analyzed by automated chip-based microcapillary electrophoresis on a Labchip GX Touch HT instrument (Perkin Elmer, Waltham, MA, USA). Amplicon sizing and relative quantitation was performed using the manufacturer's software. The interrogated alternative splicing events (ASE) in genes were statistically analyzed using one-way ANOVA and Turkey’s post hoc test.

**Quantitative real-time PCR analysis**

RNA was extracted from iPSC-CMs on day 30 or day 60 of differentiation using Trizol, and cDNA were synthesized using the Protoscript II First Strand cDNA synthesis kit protocol (NEB, Ipswich, CA, USA). qPCR assays were performed using SYBR green I detection dye on an LC480 platform (Roche, Basel, Switzerland) using the vendor’s specifications. The *SCN5A* adult isoform was amplified using the GCATACACAACTGAATTTGTGG FWD primer and the GTCTTCAGCCCTGAAATGAC REV primer. The fetal isoform was amplified using the AGTATGTCGAGTACACCTTCAC FWD primer and the CTGAAAGTTCGAAGAGCCGAC REV primer. The percentage of the adult isoform was express as a ratio calculated by dividing the adult isoform level by the sum of the both isoforms (adult + neonatal isoforms). *CACNA1C* was amplified using the TCCAGCACACCTCCTTCAG FWD primer and the AGCCCCATAAGCAGTCATCTTC REV primer. *CACNA1D* was amplified using the GCTGTTTGGCGGCAAGTTTA FWD primer and the CTTCGCCTGTCAGGATCTGG REV primer. All qPCR reactions were run in triplicate, and a non-template control (NTC) and a positive control were used in each run. qPCR efficiencies were obtained using series of cDNA dilutions and were calculated using the slope of the regression line determined using the following equation: E = 10 [–1/slope]. Only reactions with an efficiency between 1.9 and 2.1 were retained. The analysis was performed using LightCycler^®^ 480 SW 1.5 software. Run-to-run variations were adjusted using a known standard, and quantifications were corrected for efficiency. The specificity of the amplification for each run was controlled using a melting curve analysis. The housekeeping genes GATA4 (FWD primer: TCCCTCTTCCCTCCTCAAAT, REV primer: TCAGCGTGTAAAGGCATCTG) and RPL22 (FWD primer: CCATGGCTCCTGTGAAAAA, REV primer: TCACGGTGATCTTGCTCT) were used for normalization.

**Immunofluorescence staining**

iPSC-CMs were dissociated from monolayers using TrypLE Express (ThermoFisher Scientific, Saint-Laurent, QC, Canada) and were plated on Matrigel-treated 13-mm TC coverslips (Sarstedt, Saint-Léonard, QC, Canada). The cells were fixed in 4% paraformaldehyde and 4% sucrose in PBS for 15 min, washed, and permeabilized for 30 min at room temperature in 0.1% Triton X-100, 1% BSA, and 5% goat serum in PBS. The cells were incubated overnight at 4°C with the following antibodies: rabbit anti-MLC2v (1/300, Abcam, Toronto, Canada), mouse anti-cTnT (1/300, Abcam), and guinea pig anti-Na_V_1.5 (1/300, Alomone Labs, Jerusalem, Israël). The cells were then washed and were incubated for 1 h at room temperature with the following the addition of the following Alexa Fluor^®^ secondary antibodies (1/250, Life Technologies): 488 goat anti-rabbit, 594 goat anti-mouse, and 647 goat anti-guinea pig. DAPI was used to counterstain the nuclei. Cells were observed on a Zeiss LSM confocal microscope with a 63x oil objective equipped with the appropriate laser and filters.

**RNA fluorescence in situ hybridization (FISH)**

CUG-containing foci were detected in iPSC-CMs grown on coverslips using a 5’-Cy3-labeled (CAG)_5_ peptide nucleic acid probe (PNA Bio, Thousand Oaks, CA, USA) as described previously ^3^. Briefly, the cells were fixed in 4% PFA for 10 min at room temperature, washed with PBS, and permeabilized with 0.5% Triton X-100 in PBS for 10 min. The cells were washed with 2X SSC/50% formamide and were incubated with the hybridization solution (1 µg/uL yeast tRNA extract, 5% dextran sulfate, 0.3% BSA, 50% formamide, 2X SSC, 2 mM vanadyl ribonucleoside complex, 1 ng/uL peptide nucleotide probe) for 2 h at 37°C. The cells were then washed twice with 2X SSC/50% formamide for 30 min at 37°C, stained with DAPI to visualize nuclei, mounted on slides with ProLong^®^ Gold (ThermoFisher Scientific), and examined using a Zeiss LSM confocal microscope. For the visualization of cTnT, the immunostaining was performed after the FISH with additional washes with PBS between the two.

**Western blotting**

Protein extractions from hiPSC-CMs were carried out on day 30 of maturation, as described previously^27^. The western blots were performed on protein extracts from three independent differentiations for each cell line. Briefly, the differentiations were achieved in 12 wells plates. The protein extracts were made by pooling three wells together. This process was repeated on three independent differentiations (n = 3) for each cell line. Protein concentrations in the cleared lysates were determined using a DC protein assay (Bio-Rad, Saint-Laurent, QC, Canada). A standard curve was generated for each antibody to determine the linear range for the quantitative analysis. Equal amounts of total proteins (20 µg) were denatured in Laemmli 2x buffer (Sigma, St. Louis, MO, USA) for 5 min at 95°C, resolved on 4-12% gradient stain-free SDS-polyacrylamide gels (Bio-Rad), and blotted onto 0.45-µm PVDF membranes (Millipore, Etobicoke, ON, Canada). The membranes were blocked and were incubated with rabbit anti-Ca_V_1.2 (1/200, Alomone Labs) and mouse anti-GAPDH (1/1000, Cell Signaling Technology, Danvers, MA, USA) and rabbit anti-NaK ATPase pump (1/10000, Abcam). HRP-conjugated anti-rabbit antibodies were used as secondary antibodies (Jackson Laboratories). Proteins were revealed using a chemiluminescence detection kit and were visualized using a ChemiDoc MP system (Bio-Rad). Quantitative analyses were performed by normalizing the Ca_V_1.2 protein signals with the total protein signal (stain-free technology from Bio-Rad) on the membrane and were confirmed by normalization with GAPDH and NaK ATPase pump signals.

**Electrophysiology**

Patch-clamp experiments were performed at room temperature on dissociated hiPSC-CMs at days 30 to 40 post-differentiation, as described previously^4,5^. Currents and voltages were recorded in whole cell configuration using an Axopatch 200 amplifier and pClamp software (Molecular Devices, San Jose, CA, USA). Pipettes were made from fire-polished 8161 corning borosilicate glass capillaries. The liquid junction potential was not corrected. For the voltage–clamp experiments, the pipettes were coated with HIPEC (Dow-Corning, Midland, MI, USA) to minimize electrode capacitance. Macroscopic currents were recorded in spontaneously beating iPSC-CMs using the whole-cell configuration of the patch clamp technique. For I_Na_ measurements, the patch pipettes were filled with a solution composed of (in mM): 105 CsF, 35 NaCl, 10 EGTA, and 10 HEPES. The pH was adjusted to 7.4 with 1 N CsOH. The external solution was composed of (in mM): 105 NMDG, 35 NaCl, 2 KCl, 1.5 CaCl_2_, 1 MgCl_2_, 10 D-glucose, 10 HEPES, 10 TEA-Cl, and 0.5 nimodipine. The pH was adjusted to 7.4 with 1N methanesulfonic acid (MSA). For I_CaL_ measurements, the internal solution was composed of (mM): 25 NaCl, 105 CsCl, 1 MgCl_2_, 5 Na_2_-ATP, 10 EGTA, and 10 HEPES. The pH was adjusted to 7.2 with 1N CsOH. The external solution used was composed of (mM): 100 NaCl, 5 CsCl, 5 CaCl_2_, 40 NMDG, 1 MgCl_2_, 10 D-glucose, 10 HEPES. and 15 TEA-Cl. The pH adjusted to 7.4 with 1N methanesulfonic acid (MSA).

For the current-clamp experiments, APs were recorded in spontaneously beating iPSC-CMs. The membrane voltage was maintained at –80 mV: a holding current was injected during current-clamp recording to maintain the iPSC-CM resting potential near the target value. APs were elicited using 200 to 1500-pA rectangular 3-ms current pulse injections at various frequencies. The patch pipettes (resistance 5-7 mΩ) were filled with an intracellular solution composed of (in mM) 10 NaCl, 122 KCl, 1 MgCl_2_, 1 EGTA, and 10 HEPES. The pH was adjusted to 7.3 using 1 N KOH. The external solution was composed of (in mM) 154 NaCl, 5.6 KCl, 2 CaCl_2_, 1 MgCl_2_, 8 D-glucose, and 10 HEPES. The pH was adjusted to 7.3 using 1 N NaOH. Action potentials analysis were achieved considering the cell subpopulations^6^. There were two criteria to distinguish the cells subpopulation, the duration of the action potential (APD) and the shape of the action potential (the presence or no of a plateau). The criterion to distinguish the profiles of ventricular like cells was the duration of the action potential at 90 % of repolarization (APD90) when stimulated at 1 Hz. The cells with an APD90 longer than 250 ms were automatically classified as ventricular-like and the other cells (APD90 shorter than 250 ms) were classified as atrial or nodal-like. To distinguish atrial-like from nodal-like cells, the criterion was the difference between the APD50 and APD20 when stimulated at 1 Hz. Cells with a difference smaller than 10 ms (APD50 – APD20 **<** 10ms) were classified as nodal-like and the others as atrial-like. This last criterion allows to highlight the distinctive shape of the action potentials from nodal-like cells. These criteria were evenly applied through the three groups (CTRL, DM1-300 and DM1-1300) of this study to classify the action potential recorded.

Currents and voltages were recorded using an Axopatch 200 amplifier and pClamp software (Molecular Devices). Currents were filtered at 2 kHz, digitized at 10 kHz and stored on a computer equipped with an analog-to-digital converter Digidata 1300 (Molecular Devices). The data were analyzed using Clampfit (Molecular Devices) and custom-written MATLAB programs (MathWorks Inc. Natick, MA, USA).

**Optical mapping of iPSC-CMs monolayers**

Optical mapping techniques were used to study the conduction velocity on iPSC-CMs monolayers. To obtain consistent results, the iPSC-CMs monolayers were reconstituted with the same number of cells over the experiments. iPSC-CMs were dissociated at days 12 to 15 of maturation with STEMdiff™ Cardiomyocyte Dissociation Medium (STEMCELL Technologies). The monolayer was then reconstituted with 350 000 cells seed on a 13 mm TC coverslip (Sarstedt). The medium was replaced every two days for at least 15 days before experiments begin. One hour before optical recording, the iPSC-CMs (30-60 days of maturation) were feed with fresh medium. The cells were then washed, loaded with recording solution (in mM: 154 NaCl, 5.6 KCl, 2 CaCl_2_, 1 MgCl_2_, 8 D-glucose, and 10 HEPES; pH 7.3) containing 10 µM of di-4-ANEPPS voltage-sensitive dye (Thermo Fisher Scientific) and incubated for 15 minutes (37^o^C, 5% CO_2_). The cells were washed with the recording solution and kept in the incubator for 10 minutes before the recording. The membrane voltage was mapped with a high-speed CMOS N256 camera (MiCAM03, Brainvision, SciMedia Ltd, U.S.A) at a spatial resolution of 256 x 256 pixels, field of view of 16mm using 1X objective and a temporal resolution of 500 frames/sec. The imaging system includes a 530 nm green LED light source (LEX2-LZ4-G) with a stable intensity of 360 mW/cm^2^, led by an optical fiber and followed by a collimator, then a dichroic mirror (560nm) and long pass emission filter (600nm) for di-4-ANEPPS, as well as a lens system with a maximum aperture of f/1.4. The recordings were performed at 37^o^C in a microincubator (PDMI-2, Havard Apparatus). Bipolar platinum/iridium electrodes positioned at the lower edge of the preparation were used to pace the monolayer with a stimulus generator (STG4002, Multichannel Systems) at cycle of 500 ms to 2000 ms using a 10 ms current pulse duration. Optical action potentials were analyzed using Brainvision Ana software to calculate the conduction velocities and generate the maps.

**Supplementary Figures**

Supplementary Figure S1


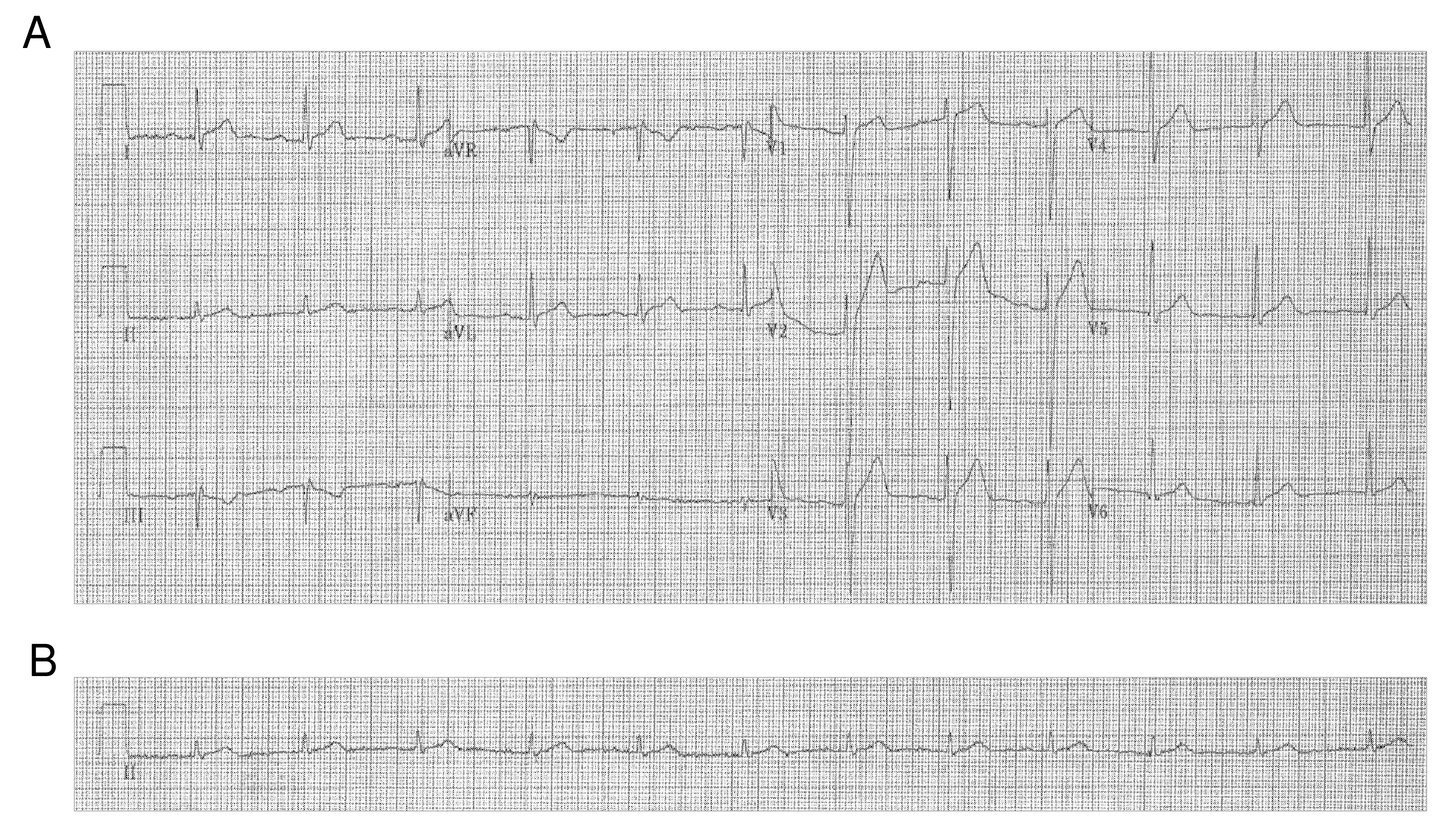


**Figure S1**: **ECG recorded in the DM1-1300 patient.** (**A**) Twelve-lead ECG from the DM1-1300 patient showing a first-degree AV block (PR interval of 212 ms), with a normal QT (QT/QTc=368/402 ms) and a normal QRS duration (98 ms). (**B**) The rhythm strip shows a first-degree AV block. The paper speed was 25 mm/s.

Supplementary Figure S2


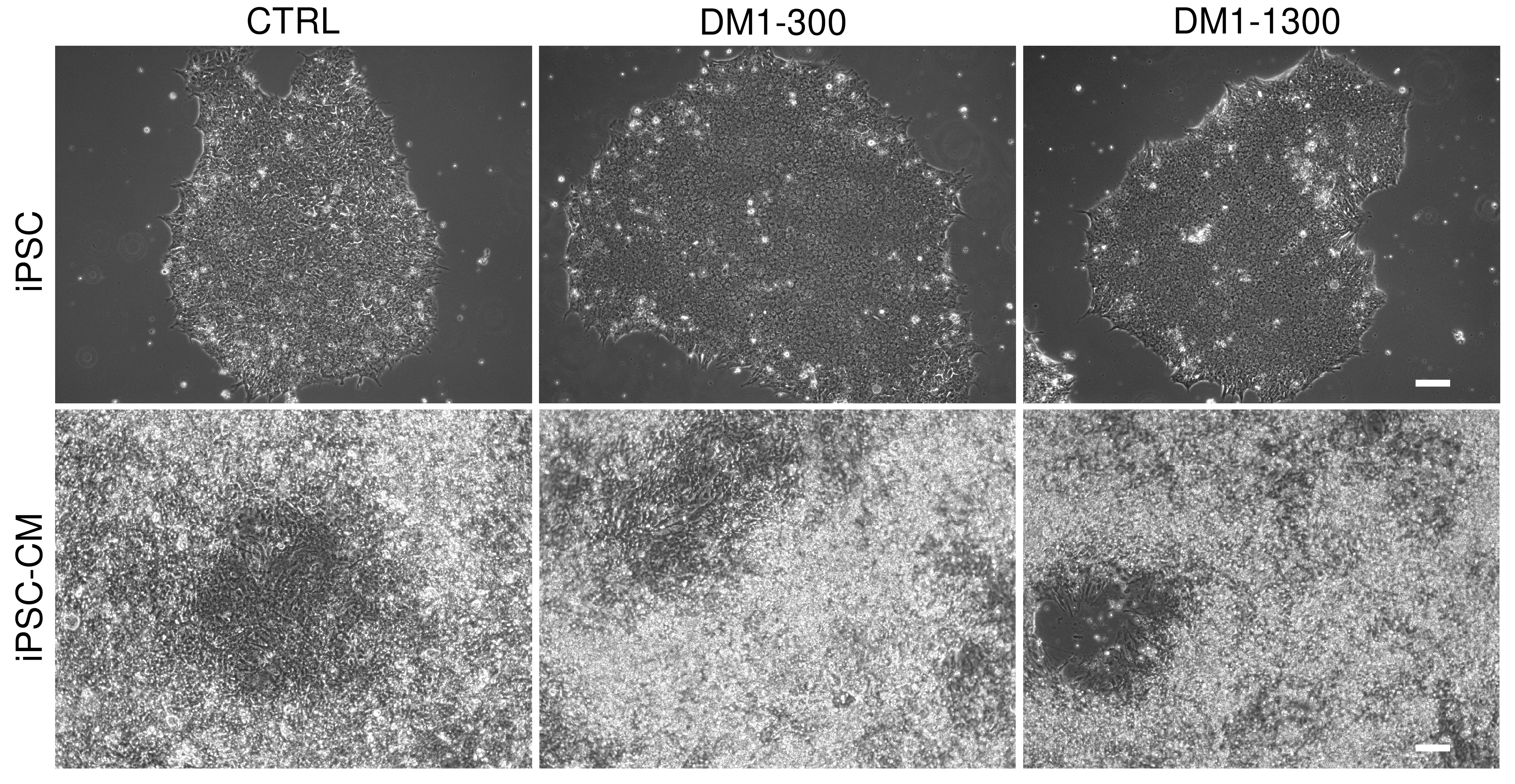


**Figure S2: Differentiation of iPSC to iPSC-CM.** Examples of iPSC colonies (top panels) and iPSC-CM after 30 days of maturation (bottom panels) from CTRL, DM1-300 and DM1-1300. Scale bar: 100 µM.

Supplementary Figure S3


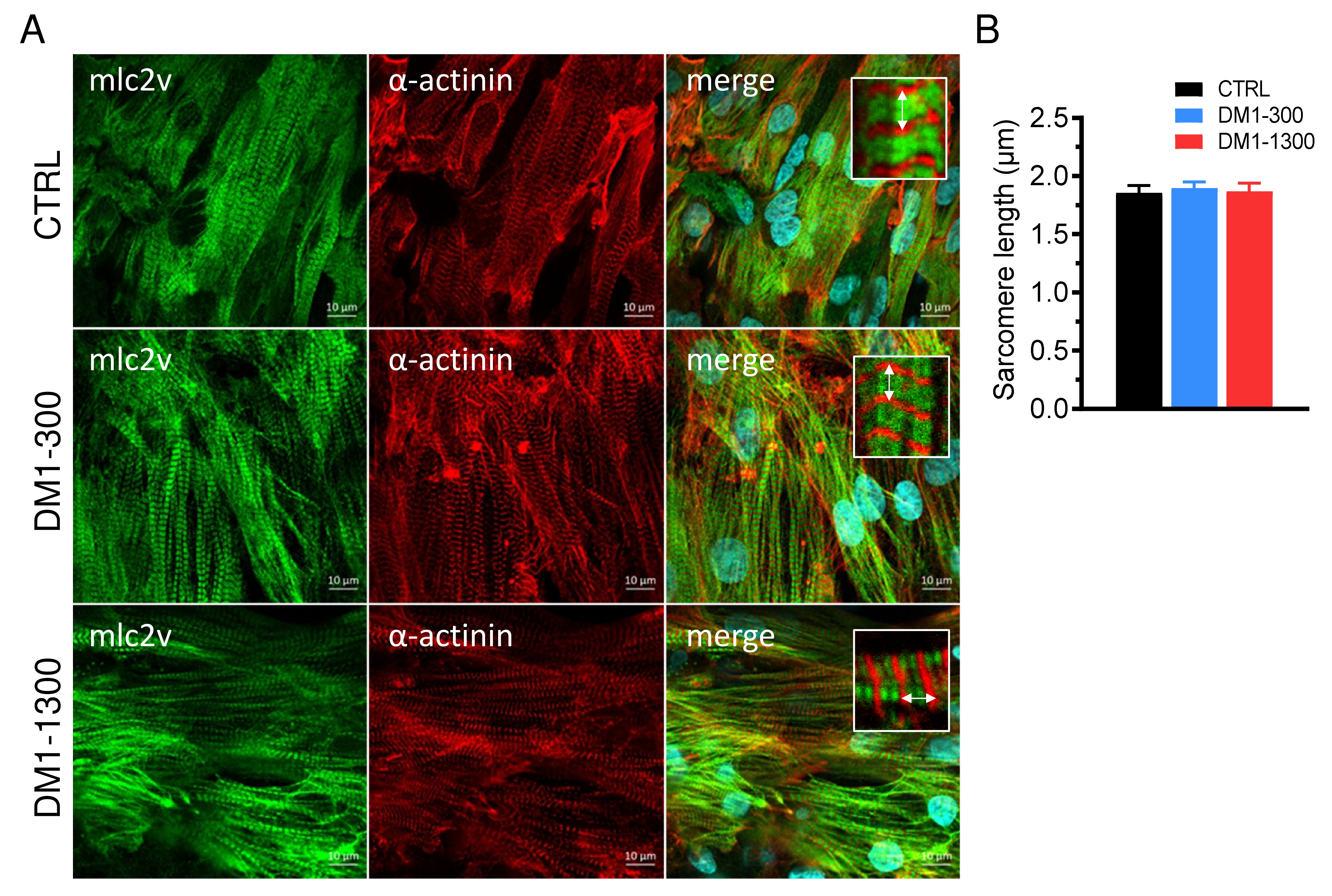


**Figure S3: Sarcomeric length measurement.** (**A**) Immunofluorescence staining of mlc2V (green) and α-actinine (red) revealed the sarcomeric structures in iPSC-CMs from CTRL, DM1-300 and DM1-1300. The last column shows merged images and the inset a zoom-in of the sarcomeres. (**B**) The sarcomeric length was measured and similar in all experimental groups. The measure was taken by calculating the distance between two α-actinine segments as indicated by the arrows.


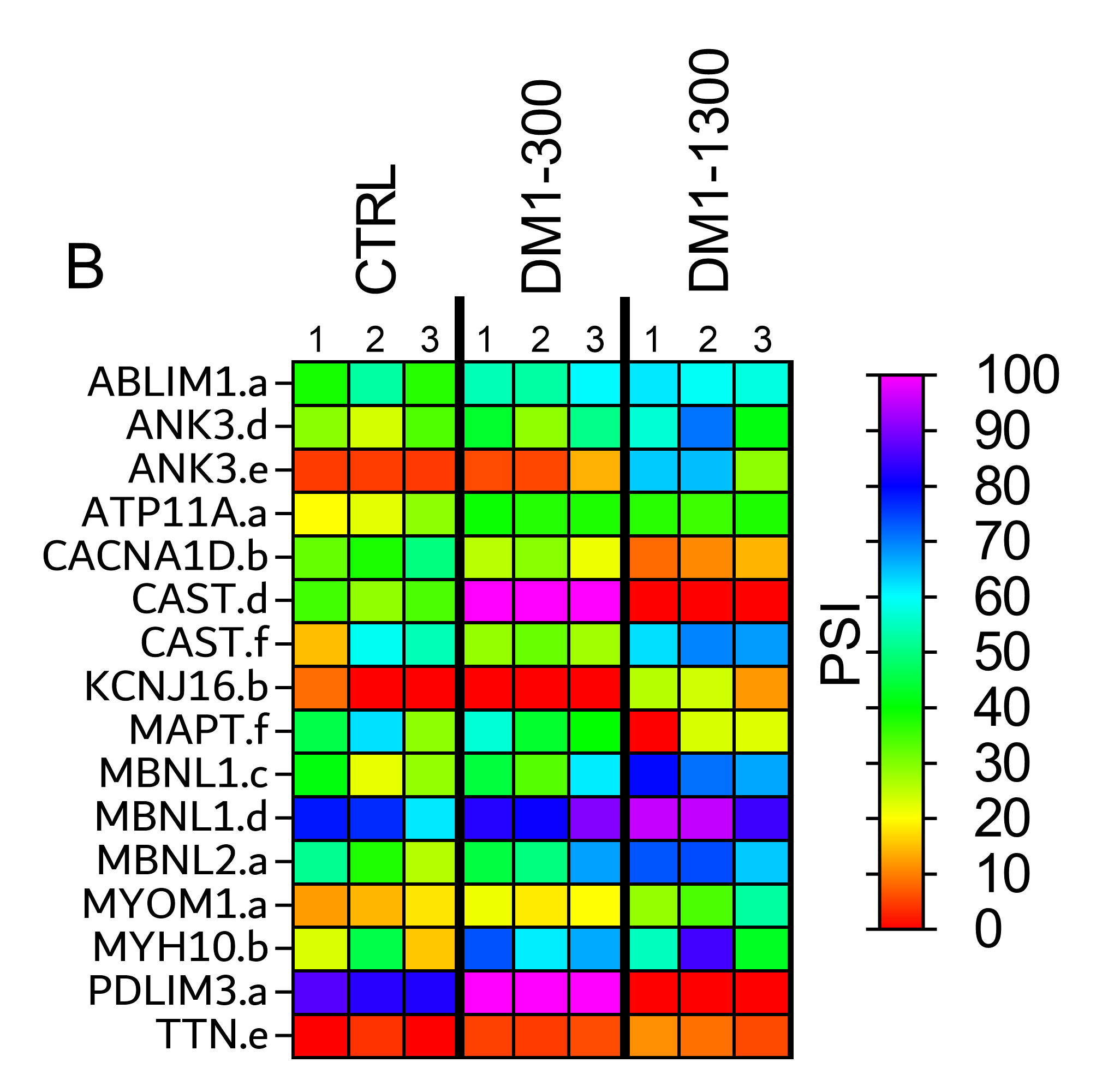

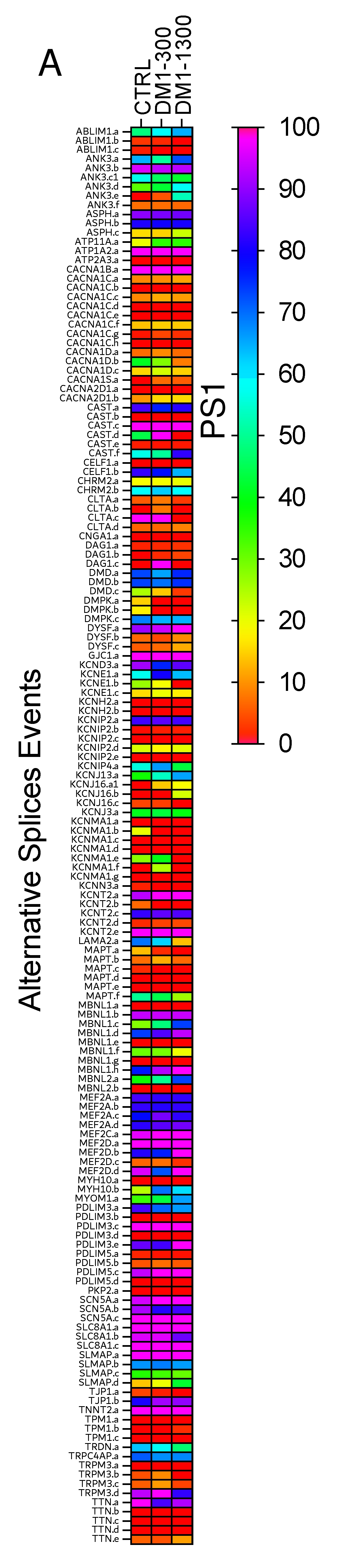
 Supplementary Figure S4

**Figure S4: The ASPCR analysis revealed splicing defects in iPSC-CMs from DM1 patients.** (**A**) Heatmap showing 154 alternative splicing events (ASEs) in 54 genes obtained from an ASPCR analysis of iPSC-CMs. This first wave of ASPCR analysis was achieved with pooled RNA extracts, namely, three RNA samples from each group (CTRL, DM1-300, and DM1-1300) from three independent differentiations were pooled before performing the cDNA synthesis (**B**) Heatmap showing 16 ASEs in 13 RNA that were significantly different between CTRL and DM1-300 and/or DM1-1300 iPSC-CMs. These results came from a second wave of ASPCR performed on three samples (1, 2, 3) from three independent differentiations for each group. They were identified using one-way ANOVA and Turkey’s post hoc test. The right panels in A and B shows the color code representing the level of splice expression as a percentage of the splice index (PSI) calculated using the following formula: longer isoform (inclusion)/sum of shorter isoform (exclusion), in %. Low and high expression levels are represented in red and purple, respectively.

Supplementary Figure S5


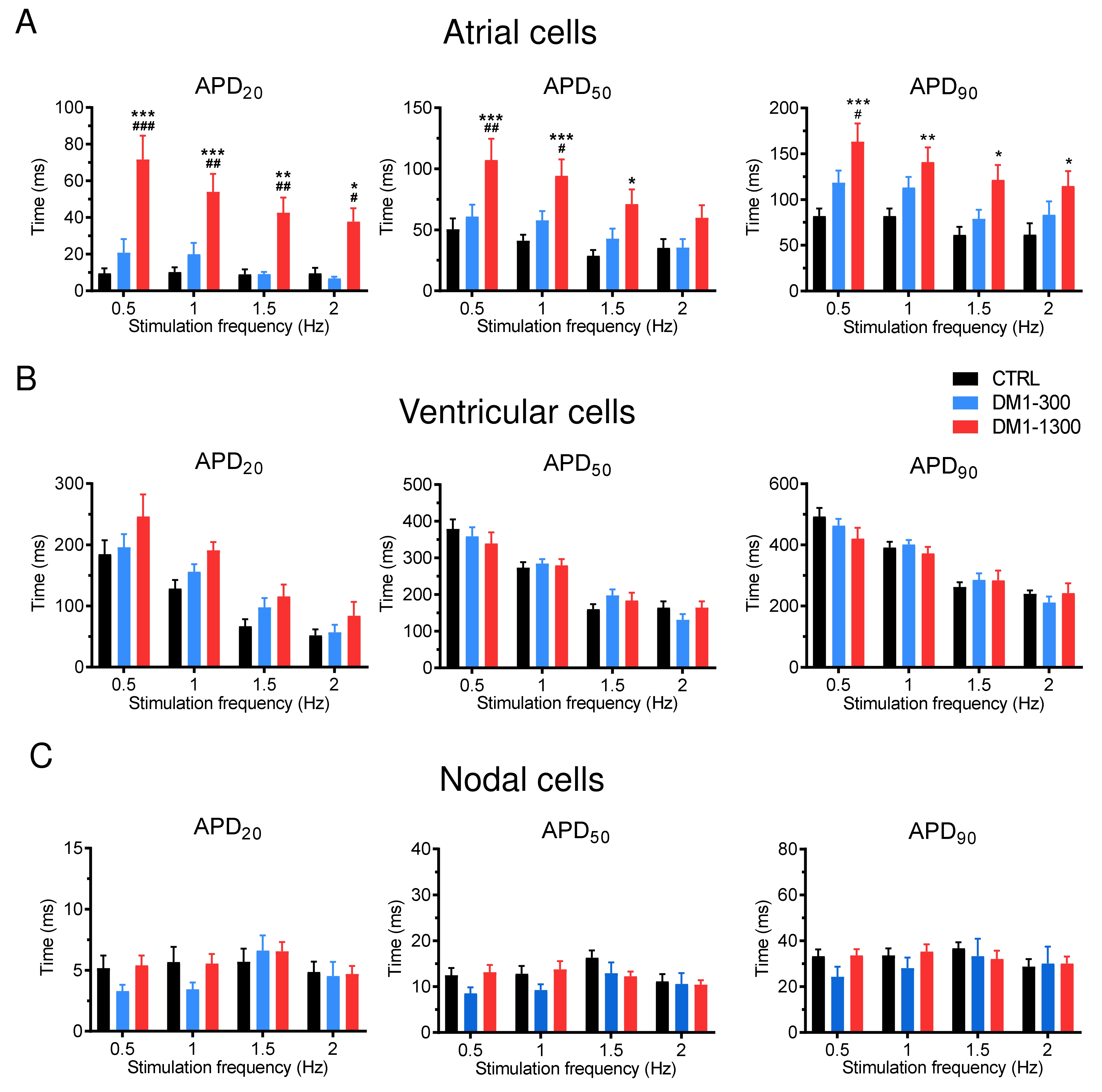


**Figure S5: Histograms summarizing the APD at 20%, 50%, and 90% repolarization at several stimulation frequencies.** APD_20_, APD_50_ and APD_90_ in (**A**) atrial-like (n = 11-21), (**B**) ventricular-like (n = 4-25), and (**C**) nodal like (n = 6-21) cells at stimulation frequencies of 0.5, 1, 1.5, and 2 Hz. Bars indicate SEM. ∗p<0.05, ∗∗p<0.01, ∗∗∗p<0.001 (CTRL vs DM1-1300) and #p<0.05, ##p<0.01, ###<0.001 (DM1-300 vs DM1-1300) as determined by ANOVA and Turkey’s post hoc test.

Supplementary Figure S6


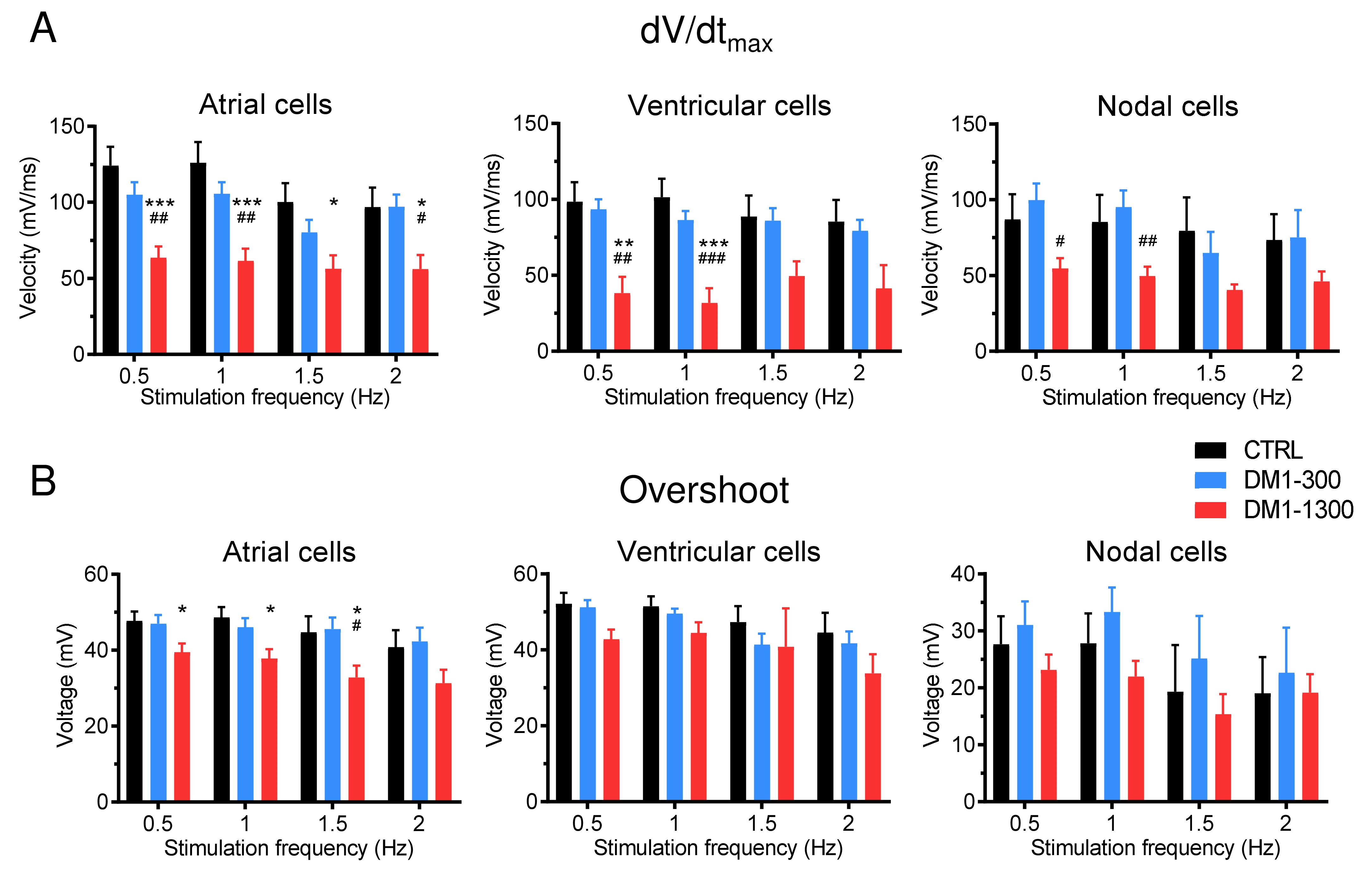


**Figure S6: Histograms summarizing the dV/dt_max_ and overshoot of APs.** (**A**) Maximal upstroke velocity (dV/dt_max_) and overshoot (**B**) of APs recorded in atrial-like (n = 12-23), ventricular-like (n = 5-25), and nodal-like (n = 6-21) cells at stimulation frequencies of 0.5, 1, 1.5, and 2 Hz. Bars indicate SEM. ∗p<0.05, ∗∗p<0.01, ∗∗∗p<0.001 (CTRL vs DM1-1300) and #p<0.05, ##p<0.01, ###<0.001 (DM1-300 vs DM1-1300) as determined by ANOVA and Turkey’s post hoc test.

Supplementary Figure S7


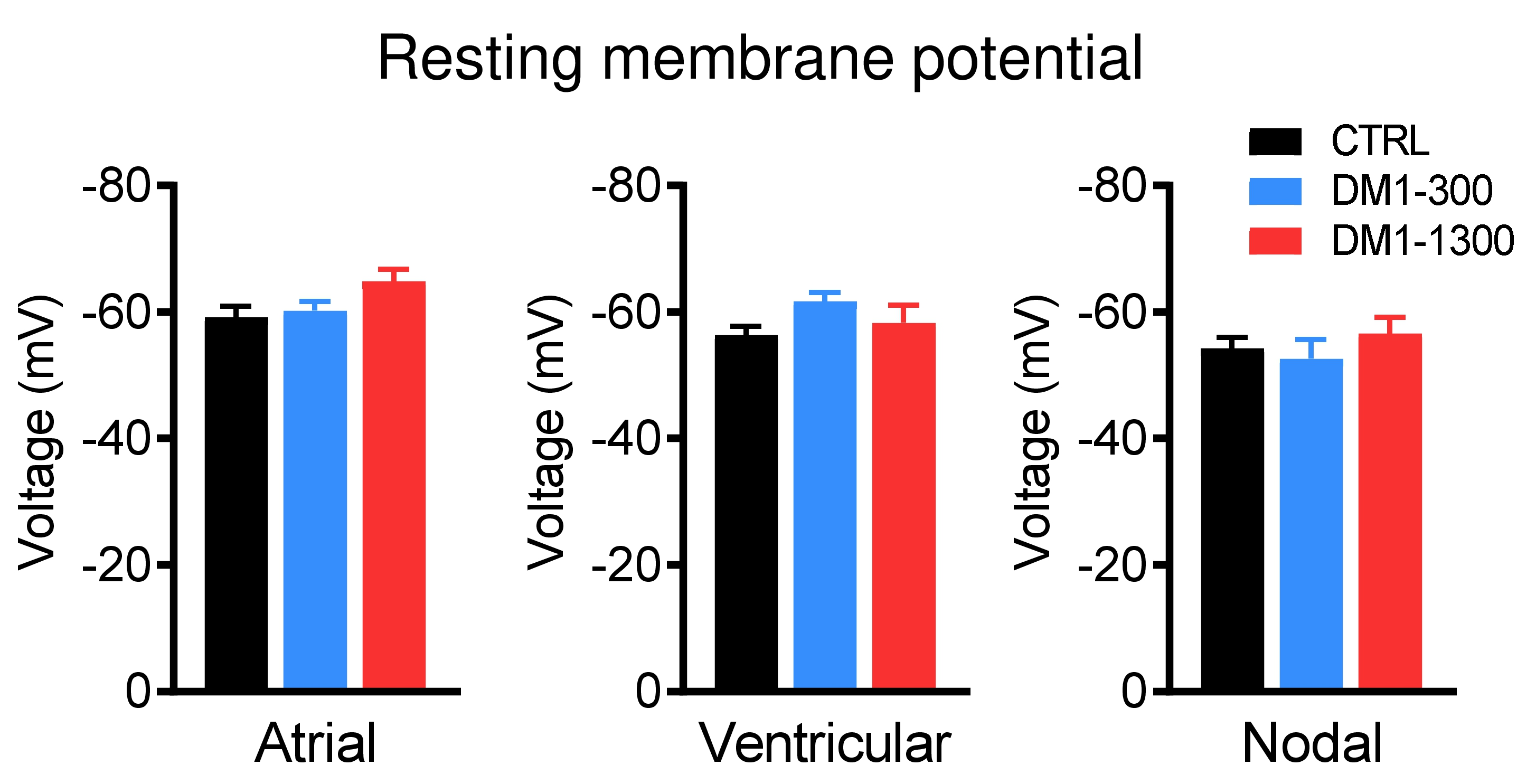


**Figure S7: Histograms summarizing the resting membrane potential.** Resting membrane potential recorded in iPSC-CMs in gap-free mode in atrial-like (n = 17-21), ventricular-like (n = 12-47) and nodal-like (n = 9-16) cells. No significative difference was observed as determined by ANOVA.

**References**

1. Burridge, P. W. *et al.* Chemically Defined and Small Molecule-Based Generation of Human Cardiomyocytes. *Nat. Methods* **11**, 855–860 (2014).

2. Lian, X. *et al.* Robust cardiomyocyte differentiation from human pluripotent stem cells via temporal modulation of canonical Wnt signaling. *Proc. Natl. Acad. Sci.* **109**, E1848–E1857 (2012).

3. Pandey, S. K. *et al.* Identification and Characterization of Modified Antisense Oligonucleotides Targeting DMPK in Mice and Nonhuman Primates for the Treatment of Myotonic Dystrophy Type 1. *J. Pharmacol. Exp. Ther.* **355**, 310–321 (2015).

4. Moreau, A. *et al.* Biophysical, Molecular, and Pharmacological Characterization of Voltage-Dependent Sodium Channels From Induced Pluripotent Stem Cell-Derived Cardiomyocytes. *Can. J. Cardiol.* **33**, 269–278 (2017).

5. Gosselin-Badaroudine, P. *et al.* A proton leak current through the cardiac sodium channel is linked to mixed arrhythmia and the dilated cardiomyopathy phenotype. *PLoS One* **7**, e38331 (2012).

6. Chen, Z. *et al.* Subtype-specific promoter-driven action potential imaging for precise disease modelling and drug testing in hiPSC-derived cardiomyocytes. *Eur. Heart J.* **38**, 292–301 (2017).

**
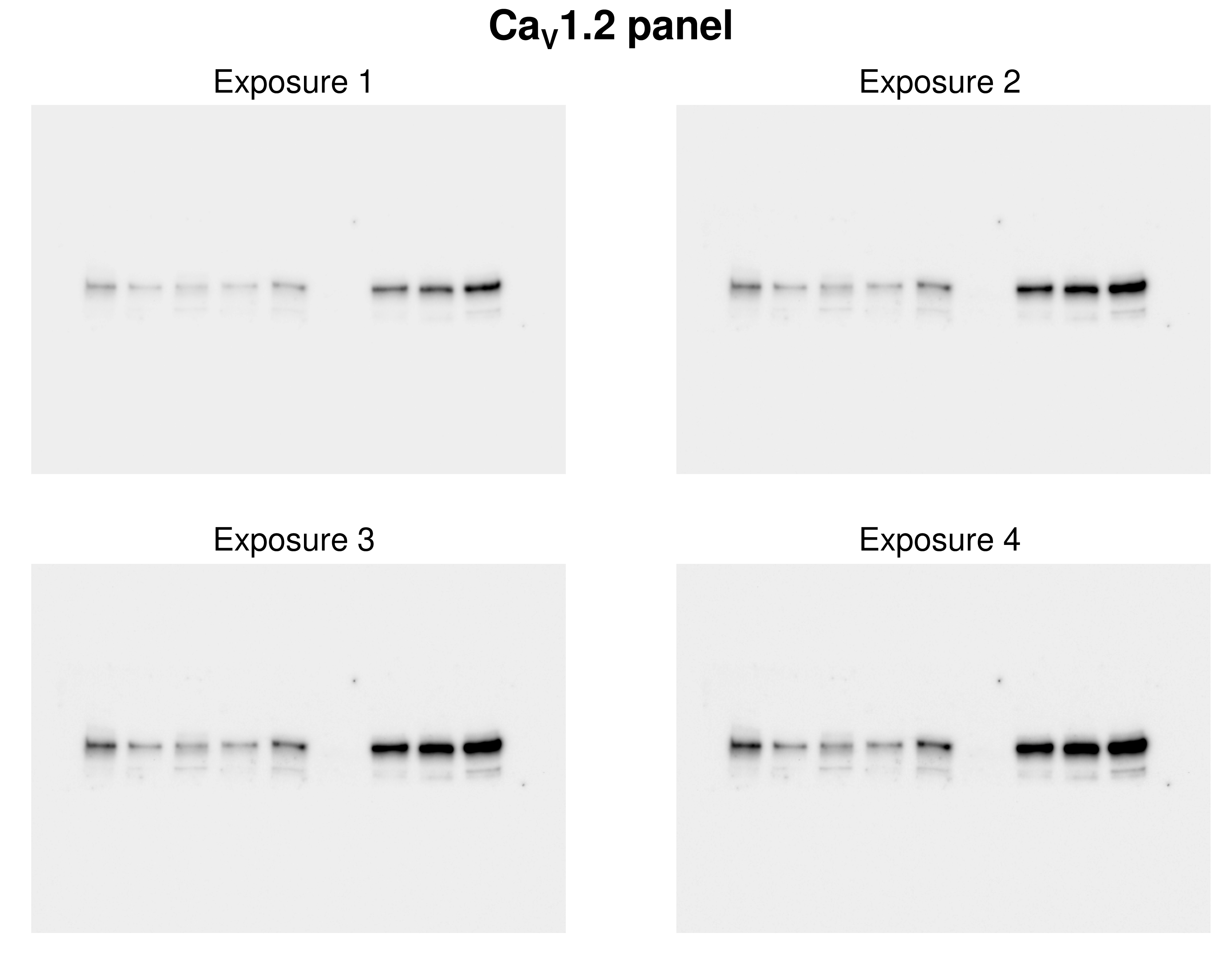
Unedited western blots images**

**B**

**A**

**
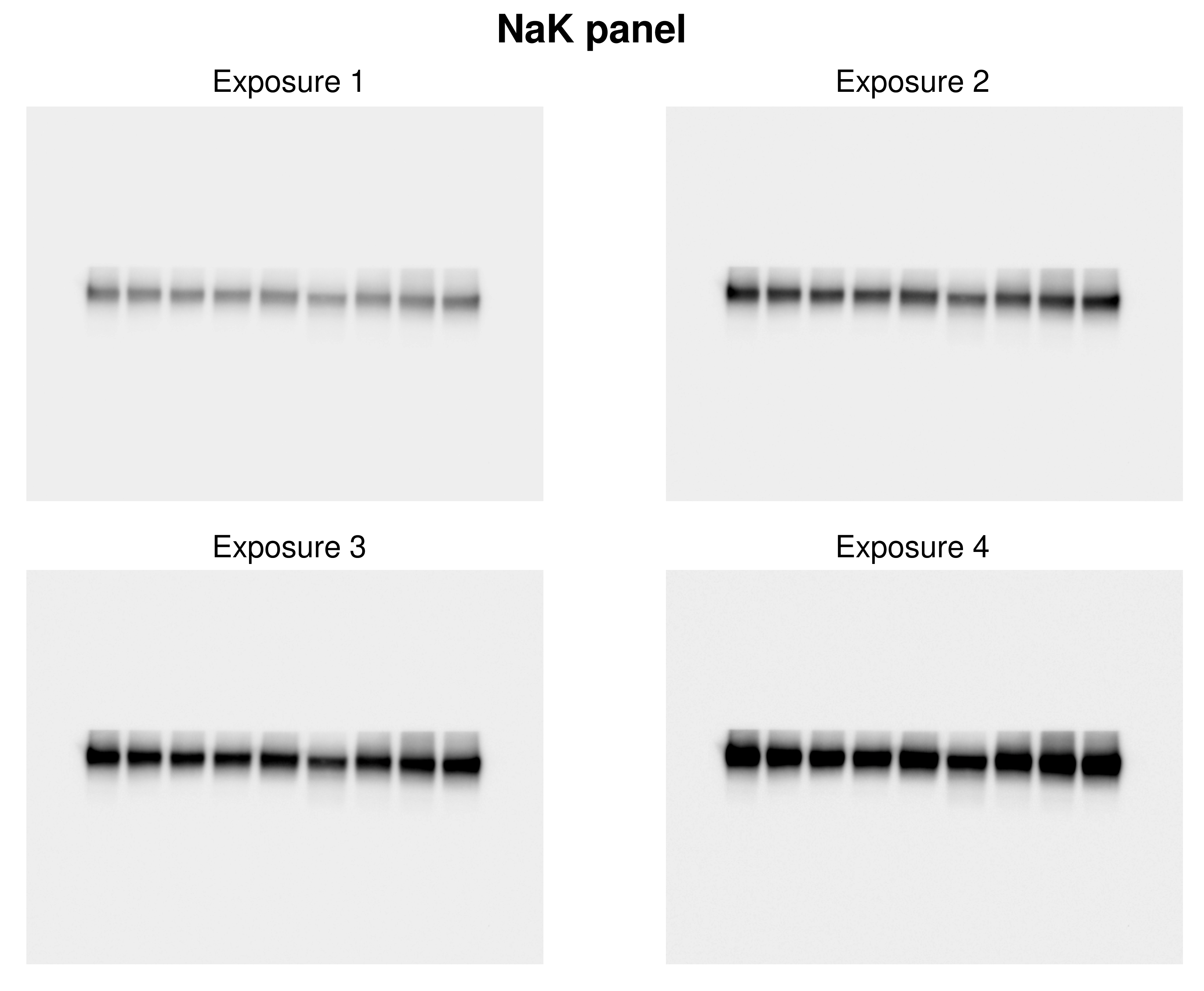
**

**D**

**C**

**
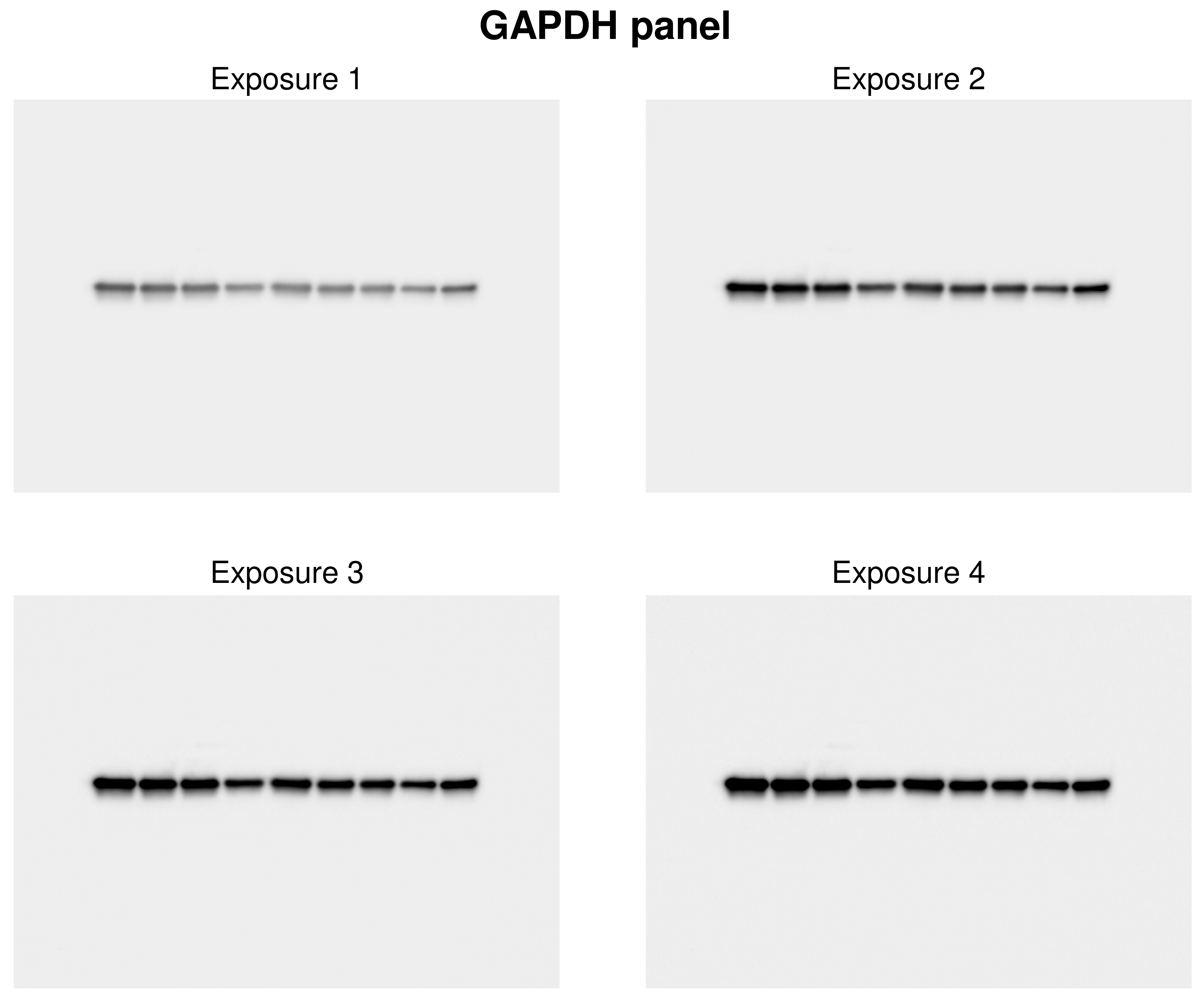
**

**
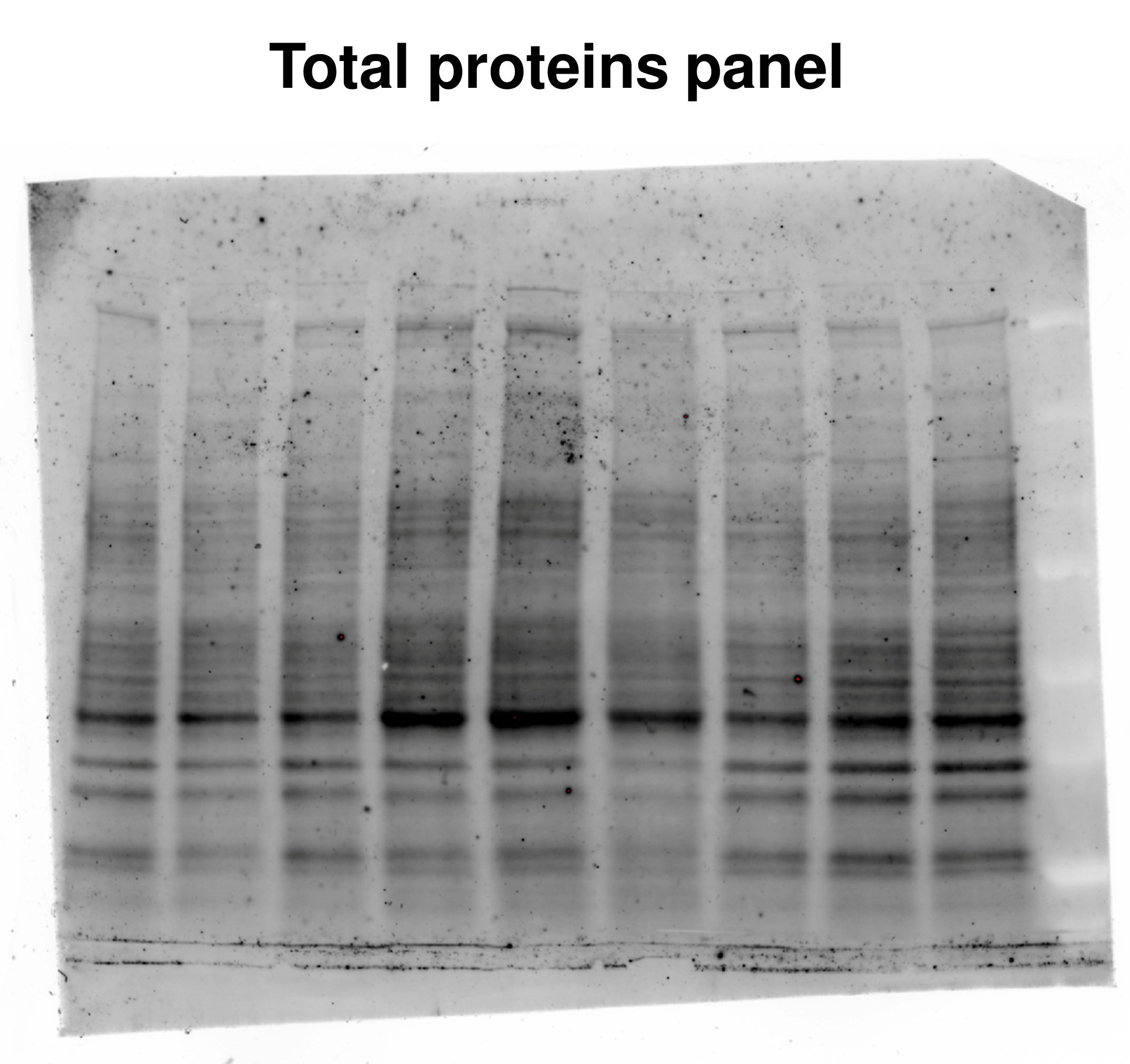
**

**Unedited blots related to Figure 4.** The Ca_V_1.2 (**A**), NaK (**B**) and GAPDH (**C**) panels come from the same transfer membrane. The membrane was cut in three parts and blotted with specific antibody. The Total proteins panel (**D**) is the membrane before it was cut and blot. The total protein was revealed using the stain-free technology form Bio-Rad (California, USA). The images were acquired with a ChemiDoc MP system (Bio-Rad).

**Supplementary Video**

**Supplementary Video 1: Cardiac conduction velocity.** Related to figure 6. Optical mapping displays showing representative action-potential propagation using di-4-ANEPPS fluorescence in iPSC-CMs from CTRL, DM1-300 and DM1-1300. The cell monolayers were paced at 1 Hz. Images were acquired using MiCAM03 imaging system (SciMedia) and processed using Ana software (SciMedia).

**Supplementary Video 2: Cardiac conduction velocity in slower motion.** Related to figure 6 and online video 1. Duplicate of the video 1 but in slower motion.
